# Increased Interferon-Induced Protein With Tetracopeptides (*IFITs*) Reduces Mycobacterial Growth

**DOI:** 10.3389/fcimb.2022.828439

**Published:** 2022-07-05

**Authors:** Abhilasha Madhvi, Hridesh Mishra, Novel N. Chegou, Bienyameen Baker

**Affiliations:** ^1^ DSI-NRF Centre of Excellence for Biomedical Tuberculosis Research; South African Medical Research Council Centre for Tuberculosis Research; Division of Molecular Biology and Human Genetics, Faculty of Medicine and Health Sciences, Stellenbosch University, Cape Town, South Africa; ^2^ Public Health Research Institute, New Jersey Medical School, Rutgers, The State University of New Jersey, Newark, NJ, United States

**Keywords:** mycobacteria, tuberculosis, IFIT, LTBI, knock-down

## Abstract

**Objectives:**

The host immune response towards *Mycobacterium tuberculosis* (*M. tb*) is known to vary with the virulence of mycobacterial species. While the majority of *M. tb-*exposed individuals develop latent TB infection (LTBI), a small proportion develops active TB disease. The milieu of understudied immune factors is believed to play an important role against host immune response towards mycobacteria. Here, we investigate the role of antiviral factors of the interferon-induced proteins with tetracopeptides (*IFITs*) family, which, in our previous research, have shown to be upregulated in response to pathogenic *M. tb*, but as yet have no established role in host response to bacterial infections.

**Methods:**

We performed vector-driven overexpression and siRNA-mediated downregulation of *IFITs* in THP-1 cells infected with different mycobacterial species. Also, we investigated the mRNA levels of *IFITs* in the LTBI and active-TB cases.

**Results:**

Overexpression of *IFITs* reduces CFUs by ~32% (30%–43%) [Median (IQR)] across three different mycobacterial strains, while knock-down increases CFUs by ~57% (41%–78%). Compared to IFN-γ, treatment of infected THP-1 cells with IFN-β significantly increases the expression of *IFITs*, while the overexpression of *IFITs* had higher mRNA expression of IFN-β than IFN-γ. Cytokines like IDO-1, IL-6, IL-23, and IFN- γ are observed to play key roles in mycobacterial survival upon *IFITs* intervention. mRNA expression levels of *IFITs* were higher in LTBI cases as compared to active TB.

**Conclusion:**

Higher expression levels of *IFITs* reduce *in vitro* survival of different drug-susceptible and drug-resistant mycobacteria and correlates with latent TB infection in infected individuals, hence emerging as an immuno-therapeutic target against *M. tb*.

## Introduction

Tuberculosis (TB) caused by *Mycobacterium tuberculosis* (*M. tb)* is a major cause of infection-related mortality worldwide and the biggest poverty-related disease. Approximately one-third of the population of the world is believed to be infected with TB (Gideon and Flynn, 2011). Of these infected cases, 5%–10% develop the disease during their lifetime, while the remaining 90%–95% are successful in containing the bacteria (Gideon and Flynn, 2011). Strikingly, most (~96%) close household contacts of TB patients remain healthy, suggesting that variations in the host immune response may determine the likelihood of active TB pathogenesis (Reichler et al., 2018).

Previous work has shown that, while the host immune response effectively restricts the growth of non-pathogenic mycobacteria, pathogenic strains of mycobacteria are often able to evade the response causing active-TB disease (Kim et al., 2018; Chai et al., 2020). Upon *M. tb* infection, different cellular antimicrobial components react to the activation of host innate immune surveillance pathways, which might be altered by *M. tb* for its advantage. A few mycobacterial factors have an inhibitory effect on host immune mechanisms (e.g., vacuolar membrane trafficking, autophagy activation, and interfering with protective Th1-type cytokine production particularly interferons), while some other factors appear to play an opposite regulator role (Chai et al., 2020).

The reason for this difference is not fully explored. In our previous work, we compared the response of human monocyte-derived macrophages to pathogenic (e.g., *M. tb*R179) and non-pathogenic mycobacteria (*M. smegmatis*) (Madhvi et al., 2020). One of the most differentially expressed gene families in our prior analysis was the interconnected family of interferon-induced proteins with tetracopeptides [*IFITs* (*IFIT1*, *IFIT2*, and *IFIT3*)], which have been previously shown to act in antiviral immunity. In the previous studies, apart from restricting viral replication, *IFIT1* and *IFIT2* were found to have important activity in LPS-induced macrophages indicating its role against bacterial infections (Berchtold et al., 2008; John et al., 2018), however, *IFITs* have never been shown to play a role in mycobacterial infections.

*IFITs* family proteins generally do not express in cells at high basal levels, but the transcription of *IFIT* genes is known to quickly increase upon viral infection or signalling of (interferon-α/β receptor) IFNAR (Sarkar and Sen, 2004). Mechanistically, *IFITs* inhibit viral protein translation, viral protein binding, and 5’-triphosphorylated and 2’-*O*-unmethylated capped RNA binding (Gebauer and Hentze, 2004; Topisirovic et al., 2011). *IFIT* genes are usually silent or expressed at low levels. *IFIT* transcription is triggered by several stimuli, mostly due to viral or bacterial infections. The strongest *IFIT* inducers are type I IFNs (IFN-α/β) and type III IFNs (IFN-λs), whereas type II IFN (IFN-γ) is much weaker (Der et al., 1998; Kohli et al., 2012). Previous studies using mouse and human cell models explain the potential of type I interferons (IFNα/β) in suppressing macrophage ability resulting in an upregulation of antimycobacterial effector molecules hence restricting the bacterial growth (*M. leprae*, *M. tb*) (de Paus et al., 2013). This effect of type I interferons was further studied using *Ifngr1* ^−/−^ *Ifnar1* ^−/−^ mice, suggesting the host protective function of type I interferons without the presence of IFNγ (Desvignes et al., 2012).

Supported by our previously published study (Madhvi et al., 2020), we hypothesized that modulation (overexpression and knock-down) of *IFITs* in THP-1 cells may counter the *in vitro* growth of mycobacteria. Our laboratory successfully patented the innovative technology of using the *IFITs* polypeptide for treating tuberculosis, which requires further human studies and clinical trials (Madhvi and Baker). Therefore, our present work is a preliminary investigation for exploring the role of *IFITs* as an important host–pathogen interaction factor during *M. tb* infection. We investigated the role of *IFITs* expression levels on the relative growth and survival of mycobacteria in human monocyte-derived macrophages and TB patient samples. Our findings demonstrate the vital role of *IFITs* against mycobacteria.

## Materials And Methods

### Human Samples

We enrolled 12 healthy participants with equal gender distribution and 24 cases (12 LTBI and 12 TB) for clinical protocol. **Supplementary Figure 1** demonstrates the stepwise methodology adopted for the experiments in the study. We obtained ethical approval from the Health Research Ethics Committee (HREC), Stellenbosch University, Tygerberg campus, Cape Town (*in vitro* infection protocol: HREC reference #S17/10/211, *clinical* protocol: HREC reference: N16/05/070).

### Culture of THP-1 Cells

THP-1 cells were used for the *in vitro* infection and intervention experiments. These cells were previously shown to be a suitable alternate of hMDMsfor the *in vitro* infection experiments with mycobacteria (Bosshart and Heinzelmann, 2016; Madhvi et al., 2019). We preferred to use THP-1 cells since they are easier to revive and more stable for *in vitro* experiments. Commercially available THP-1 cells (ATCC-88081201) [European Collection of Authenticated Cell Cultures (ECACC), Salisbury, UK] were cultured in RPMI-1640 medium supplemented with 10% heat-inactivated fetal calf serum (Biochrome, Germany). THP-1 cells were treated with a final concentration of 100 nM phorbol 12-myristate 13-acetate (PMA; Sigma Aldrich, USA) for 48 h. For infection experiments, THP-1 cells were seeded in 48-well plates (Greiner Bio-One, cat. No. 677180) with 0.07 x 10 (John et al., 2018) cells per well.

### Cytotoxicity of THP-1 Cells

We tested cell cytotoxicity using Roche Water Soluble Tetrazolium (WST-1) Cell Cytotoxicity Reagent (Roche, USA) in 1:10 dilution of WST-1 reagent to RPMI complete media (RPMI + 10% human serum). Cells were incubated for 1 h at 37°C and 5% CO_2_, and absorbance was read at 450 nm with a 630-nm of wavelength correction on a multimode reader.

### Infection With Mycobacteria

*M. tb* R179 [Beijing genotype strain R220, clinical isolate, Johnson et al. (2006)], *M*. *bovis* BCG [Pasteur 1743P2 strain, laboratory collection, Viljoen et al. (2013)], and *M. smegmatis* [MC155 strain, laboratory collection, Harper et al. (2010)] species of mycobacteria were used for infection at multiplicity of infection (MOI) of 1. Mycobacteria were cultured in 7H9 medium with added 10% OADC and 0.5% glycerol (Sigma Aldrich, St. Louis, MO, USA) without Tween 80. We avoid the use of Tween, as Tween is known to affect macrophage uptake and immune response to *M. tb* (Leisching et al., 2016).THP-1 cells were infected with each mycobacterial species at aMOI of 1 and 4 h were allowed for mycobacterial uptake.

### Quantitative RT-PCR

We processed cells (human macrophages) at 12- and 96-h post-infection for RT-PCR. Total RNA was extracted with the help of RNeasy Plus Mini Kit (Cat. No. 74134, Qiagen, Limburg, Netherlands), following manufacturer’s protocol. Good quality RNA (RIN >9, 0.8 µg in concentration) was used for cDNA preparation using the Quantitect R Reverse Transcription Kit. qRT-PCR amplification performed using a LightCyclerR 96 system (Roche, Germany). LightCyclerR 480 SYBR Green I Master was used for various differentially expressed genes using QuantiTectR primer. Hs-GAPDH and Hs-UBC as two housekeeping genes were selected as reference genes conferring stable expression levels. The amplification process involved 45 cycles of 95°C for 10 s followed by 60°C for 10 s, and finally, 72°C for 10 s. The qRT-PCR was used to access the effect of co-stimulation with IFN-α, IFN-β, and IFN-γ on expression of IFITs (**Supplementary Figure 2A**), and also the effect of overexpression of *IFITs* on the mRNA expression of IFN-α, IFN-β, and IFN-γ (**Supplementary Figure 2B**).

### Vector-Mediated Overexpression

epcDNA3.1 3xFlag was used as a vehicle vector for transfecting *IFIT1*, *IFIT2*, or *IFIT3* (**Supplementary Figure 3**). These plasmids were a gift from Kathleen Collins (Addgene plasmid # 53554;:53554; RRID: Addgene_53554) (Kamens, 2015). Transfection in THP-1 cells was achieved using Mission^®^ siRNA (Theis and Buchholz, 2010) transfection reagent and confirmed with Western blot (**Supplementary Figure 4**). The empty vector pcDNA3.1 3xFlag was used as a negative control. A titration-based plasmid concentration of 120 ng/ml for each plasmid construct of *IFIT1*, *IFIT2*, and *IFIT3* was used for transfections in 0.07 x 10 (John et al., 2018) of THP-1cells (**Supplementary Figure 5**).

### siRNA Knock-Down

Gene silencing targeting two different silencing sites for each *IFIT1*, *IFIT2*, and *IFIT3*, respectively, was using manufacturer’s protocol and was measured at 24 and 96-h post-transfection and confirmed with Western blot (**Supplementary Figure 4**). A scrambled sequence was used as a negative control siRNA (Cat. No. S103650325). **Supplementary Table 3** provides the detailed list of siRNA premix used.

Supplementary data provide details of plasmid purification, bacterial uptake, and activation of IFN-β and IFN-γ.

### Statistical Analysis

The qRT-PCR data were analysed using LightCycler 96 SW 1.1 Software (Netherland, BV) and GraphPad Prism Version 7 Software; https://www.graphpad.com/company/(GraphPad Software, San Diego California, USA). Relative expression which measures target transcript in a treatment group to that of the untreated group was measured through the software in response to the calibrator and non-transcription control. The expression was related to the control group where the calibrator was normalized to one. The significance of the effect of strain was determined by one-way ANOVA and Tukey’s honest significant difference test to correct for multiple testing.

Cytotoxicity graphs and CFUs were plotted with an average of the technical triplicates leading to the mean of all the biological replicates. The percentage of every expressing cell was generated, and *p* value was calculated using two-way ANOVA with Tukey’s correction. Luminex data were analysed by two-way ANOVA with Tukey’s correction.

## Results

### Higher Expression of IFIT in LTBI Compared to TB Cases

#### Individuals With Latent TB Infection Express Higher Levels of IFITs Than Those With Active TB

**Figure 1:**
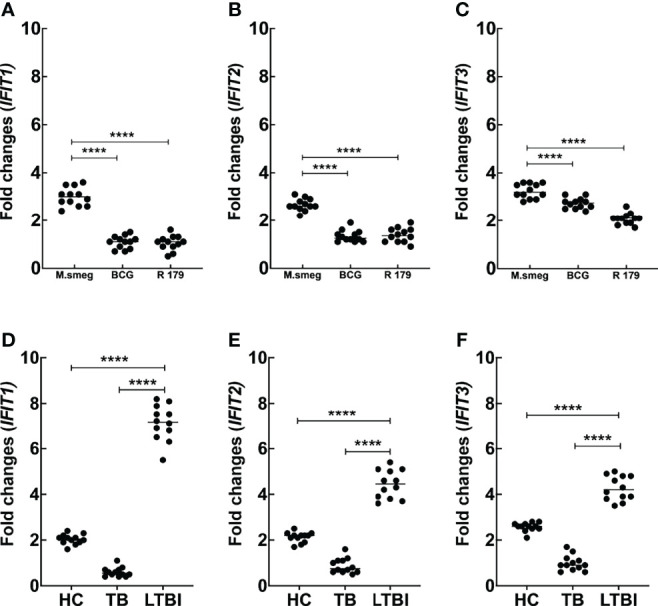
Comparison of mRNA expression (fold changes) for *in vitro* protocol [*M. smegmatis, M. bovis* BCG and *M. tb* R179] for **(A)** *IFIT1*, **(B)** *IFIT2* and **(C)** *IFIT3.* We also compared mRNA expression of *IFIT1*,**(D)**, *IFIT2* **(E)** and *IFIT3* **(F)** in clinical cohort across HC, TB and LTBI group. We observed significantly higher expression of *IFIT1, IFIT2* and *IFIT3* in LTBI cases as compared to HC and TB. This indicates role of *IFITs* in mycobacterial containment in LTBI cases. For *in vitro* protocol, we also observed higher expression of *IFITs* in species with lower pathogenicity. BCG, Bacillus Calmette-Guerin; HC, healthy control; *IFITs*, interferon-induced protein with tetratricopeptides; LTBI, latent tuberculosis infection; TB, tuberculosis **Supplementary Figures 7A–C** depicts the mRNA fold changes of different genes (20 other genes) across the Clinical and *in vitro* protocols. ****p < 0.0001.

Building on our previous work that showed significantly higher expression of *IFITs in vitro* in cells infected with pathogenic *M. tb* compared to non-pathogenic *M. smegmatis*, we first asked whether the expression level of *IFITs* may also differ between individuals with active TB (TB) versus latent TB infection (LTBI). For this analysis, we included samples from 12 participants with active TB, 12 participants with LTBI, and 12 healthy controls. The LTBI group had a higher proportion of females as compared to active TB cases, and full demographic information for the participants is available in **Supplementary Table 1**. We found mRNA expression of *IFIT1*, *IFIT2*, and *IFIT3* to be higher in THP-1 cells infected with *M. smegmatis* as compared to *M. bovis BCG* and *M. tb* R179 **[** **Figure 1 (A–C)** **]**.

Strikingly, we found that cells from active TB patients expressed the lowest levels of *IFIT1*, *IFIT2*, and *IFIT3* mRNA, whereas healthy controls had at least double the transcript level as compared to active TB patients, and LTBI participants had even higher expression levels (4-fold to 8-fold higher than active TB) **[** **Figures 1 (D–F)** **]**. These findings suggest that *IFITs* levels may be involved in controlling TB infection and raises the possibility that they may help to distinguish between active TB and LTBI.

### IFIT Expression Levels Influence Mycobacterial Growth In Infected Cells

**Figure 2:**
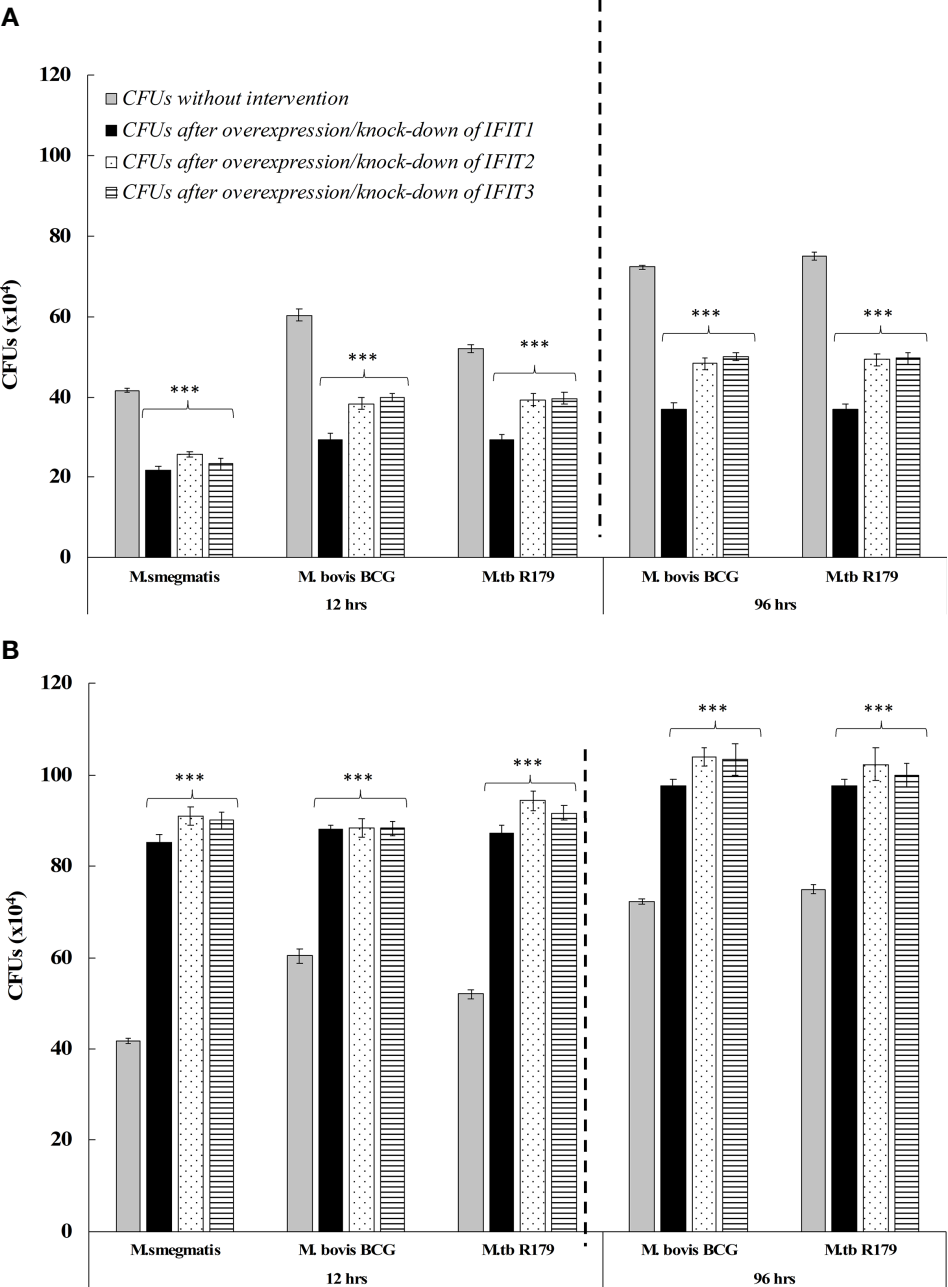
Comparison of *in vitro* CFUs of *M. bovis* BCG after overexpression of *IFITs* at 12-hours and 96-hours **(A)**; and knock-down of IFITs at 12-hours and 96-hours **(B)**. Compared to *M. bovis* BCG, we observed significant reduction of CFUs after overexpression of *IFITs* and significant increase of CFUs after knock-down of *IFITs.* The results indicate the association of *IFITs* with *in vitro* survival of Mycobacteria. BCG, Bacillus Calmette-Guerin; IFIT, interferon-induced protein with tetratricopeptide. **Table 2** depicts the %-increase or decrease in CFUs after *in vitro* knock-down and overexpression respectively. ***p<0.001.

Prompted by our findings above, we wondered whether higher expression levels of *IFITs* might protect cells against mycobacteria. In order to investigate this, we used THP-1 cells (a common *in vitro* infection model for mycobacteria) and modulated *IFIT* gene expression *via* plasmid-based overexpression or siRNA-mediated knockdown. We found a higher number of CFUs after knock-down of *IFITs*, whereas CFUs decreased considerably after overexpression of *IFITs* across all three mycobacterial species (**Figure 2**). **Supplementary Table 2** depicts the %-increase or decrease in CFUs after knock-down and overexpression respectively. Compared to untreated, we observed an overall 32.5% reduction in CFUs after overexpression of *IFIT1*, *IFIT2*, and *IFIT3* across three species. Similarly, an overall 57% increase in CFUs was observed after knock-down of *IFITs.* These results indicate the importance of *IFITs* role in mycobacterial survival. The viability of the THP-1 cells was high (>85%) and consistent across uninfected and mycobacteria-infected conditions (**Supplementary Figure 6**). Also, the comparison of bacterial uptake by THP-1 cells across different mycobacterial species without intervention, after overexpression and knock-down of IFITs were found to be similar (**Supplementary Figure 7**).

### IFIT Expression Levels Influence a Broad Cytokine Profile

Given the marked effect of *IFIT* expression on mycobacterial CFUs and the observation that individuals with stable control of latent TB infection have high *IFIT* levels, we inquired whether *IFIT* expression may induce a broader cellular program that is protective against mycobacteria. To investigate this further, we quantified the expression of a panel of cytokines in the same patient-derived cells and THP-1 cells described above. This panel was carefully selected based on their differential expression found in our previous study (Madhvi et al., 2020). For the patient-derived cells, we found (by qRT-PCR) that mRNA expression of IFN-α, IL-6, IL-8, IL-12β, ISG-15, MX1, MX2, and RSAD2 was lower, while the expression of IL-1β, IL-4, IDO-1, IFN-γ, IFI44, IFI44L, MT1A, TRIB3, and TNF-α was higher in LTBI cases compared to healthy controls and active TB cases (**Supplementary Figures 8A–C**). Overexpression of IFITs led to an increase in the levels of many cytokines including Interferon and Interleukin family, whereas knockdown of *IFITs* had the opposite effect (**Supplementary Tables 4–6**).

**Figure 3:**
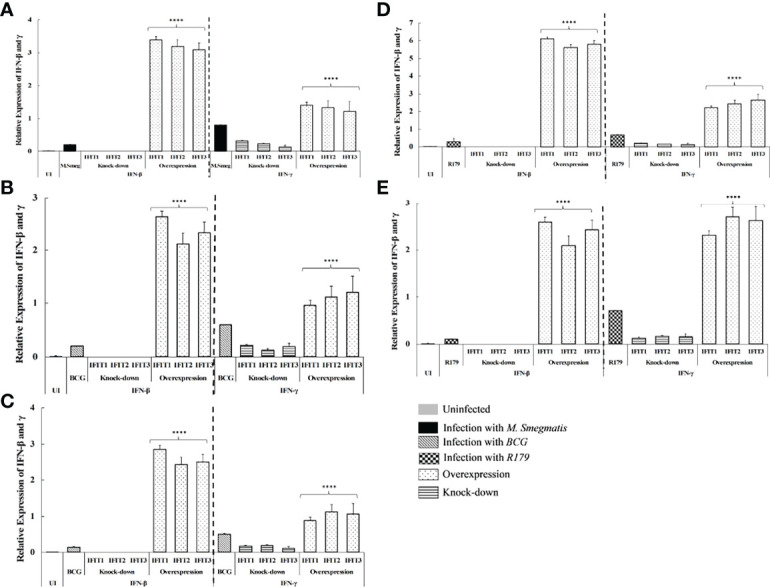
qRT-PCR determining the effect of overexpression and knock-down of *IFITs* on expression of IFN-β and IFN-γ in hMDMs infected with **(A)** *M. smegmatis* at 12 hours, *M. bovis* BCG at **(B)** -12 hours, **(C)** -96 hours, *M. tb* R179 at **(D)** -12hours and **(E)** -96 hours post infection. Compared to untreated CFUs and knock-down, we found higher expression of IFN-β and IFN-γ in *IFITs* overexpressed hMDMs. This indicates the association of these two cytokines with reduction of CFUs after knock-up vs. knock-down). ***p<0.0001 (comparison between knock-up vs. knock-down).

**Figure 4:**
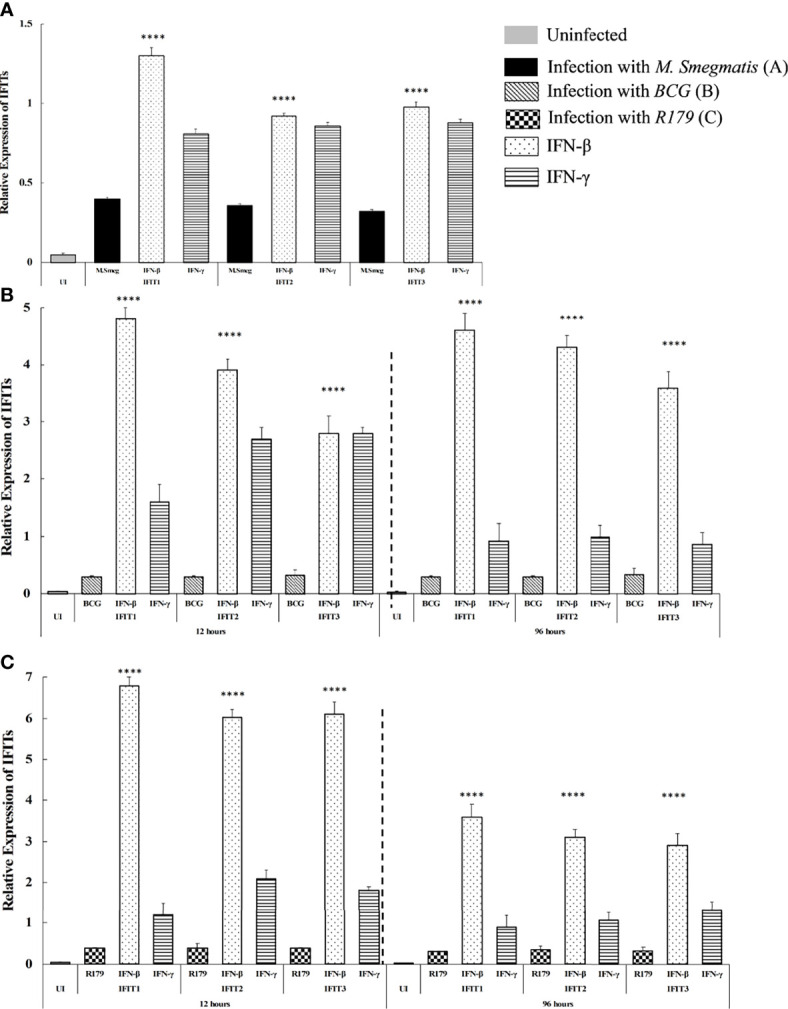
qRT-PCR determining the effect induced from treatment of IFN-β and IFN-γ on the expression of *IFITs* in hMDMs infected with **(A)** *M. smegmatis* at 12 hours, **(B)** *M. bovis* BCG at -12 hours and -96 hours, and **(C)** *M. tb* R179 at -12hours and -96 hours post infection. In hMDMs after infection with all three species (*M smegmatis, M bovis* BCG and *M.tb* R179), we found expression of *IFIT1, IFIT2 and IFIT3* found to be higher in hMDMs stimulated with IFN-β compared to untreated and IFN-γ treated hMDMs. ****p<0.0001 (higher in IFN-β stimulated hMDMs).

It is noteworthy that compared to untreated CFUs and knock-down, we found higher mRNA expression of IFN-β and IFN-γ in IFITs knocked-up hMDMs. This indicates the association of these two cytokines with reduction of CFUs after overexpression of IFITs (**Figure 3**). Since IFN-γ is known to provide protective immunity against intracellular pathogens primarily by activating macrophage, we therefore speculate its role in the modulating *in vitro* Mtb CFU counts after knock-down/overexpression of IFITs. On the other hand, the expression of IFITs was found to be significantly higher in IFN-β treated THP-1 cells compared to IFN-γ treated cells (**Figure 4**), indicating that increase of IFITs after induction from IFN-β is protective against mycobacteria. Although, we do not observe a significant change in M. smegmatis (a non-pathogenic strain of mycobacteria) here, the difference observed in other two pathogenic strains depict a protective mechanism against *M. tb.*

## Discussion

In this study, we profiled the expression level of *IFITs* in patient-derived cells from LTBI, TB, or healthy controls, and investigated the effect of *in vitro* overexpression or downregulation of *IFITs* on the survival of mycobacterial species and cytokine expression profiles in infected THP-1 cells.

The following are the main results: (a) *in vitro* overexpression of *IFITs* found to reduce ~32% (30%-43%) [Median (IQR)] of mycobacterial CFUs; (b) *in vitro* knock-down found to increase ~57% (41%-78%) [Median (IQR)] of CFUs; (c) both mRNA expression and cytokine levels significantly increase after overexpression, and reduce after knock-down of IFITs in *M. smegmatis* infected cells compared with *M. bovis* BCG and *M. tb* R179; (d) mRNA expression of key cytokines found to be higher in LTBI cases compared to active-TB cases; (e) overexpression of *IFITs* increase the mRNA expression of IFN-β compared to IFN-γ; (f) THP-1 cells treated with IFN-β expressed higher levels of all *IFITs* as compared to IFN-γ treated cells; and (g) pro-inflammatory cytokines such as IDO-1, IL-6, IL-23, and IFN-γ found to increase or decrease significantly upon *IFITs* intervention.

We observed a vice versa association between an increase in *IFITs* expression and increased expression (mRNA and protein levels) of IFN-β as compared to IFN-γ. These results reaffirm the importance of induction of IFN-β against *M. tb*, as hinted by previous studies (Sabir et al., 2017). However, it is important to note that this induction maybe indirect, such as *via* host mitochondrial DNA. A previous study by Wiens et al. highlighted that host mitochondrial DNA (mtDNA), not mycobacterial DNA, contributes to IFN-β induction (Wiens and Ernst, 2016). However, another study showed IFN-β as a down regulator of host immune responses against *M. tb* (Sabir et al., 2017). The anti-mycobacterial roles of IFN-β include its ability to downregulate IL-1β and IL-18 and upregulate IL-10 (Sabir et al., 2017). IFN-β signalling pathway, through the STAT1, suppresses the expression of the inflammasomes (NLRP1 and NLRP3), foremost to the suppression of caspase-1-dependent IL-1β maturation (Sabir et al., 2017). IFN-γ is primarily responsible for activation of macrophages and bactericidal activity (Reljic et al., 2010). Previous studies also demonstrated that *M. tuberculosis* regulates host IL-6 production to inhibit type I interferon and, consequently, disease progression (Martinez et al., 2013). IL-23 have been demonstrated to confer protective cellular responses and promote survival against *M. tuberculosis* (Cooper et al., 2007).

*IFITs* are known antiviral proteins which provide immunity against viral infections. These proteins are predominant in vertebrates while they also share their homology in several other organisms. IFIT protein production is a result of interferon treatment and viral infections (Varela et al., 2014). The mechanism of *IFITs* in preventing viral infection is by sequestering and regulating the functions of RNAs and the viral proteins (Fensterl and Sen, 2014). However, the role of *IFITs* in bacterial infections has also been reported. One previous study with LPS-activated macrophages and siRNA screen identifies *IFIT1* as negative regulator of pro-inflammatory genes and positive regulator of interferon stimulated genes (ISGs) (John et al., 2018). Another study demonstrated that activation of macrophages with LPS or IFN-γ resulted in the expression of *IFIT2* in a type I interferon-dependent manner (Berchtold et al., 2008). The study further showed that LPS increases the expression of TNF-α, IL-6, and MIP-2 but not of *IFIT1 (* Berchtold et al., 2008 *).* However, *IFIT*’s role remains understudied in the mycobacterial response of immune cells including macrophages.

Our results showed that *IFITs* play an important role in limiting mycobacterial survival. IFN-β was not detected in the THP-1 cells infected with different mycobacterial species without intervention. But thelevels of IFN-β (mRNA and protein) significantly increase upon overexpression of *IFITs* while reducing the corresponding CFUs, indicating that *IFITs* modulate *in vitro* survival of mycobacteria *via* IFN-β.Pro-inflammatory cytokines such as IDO-1, IL-6, IL-23, and IFN-γ are also observed to alter significantly upon *IFITs* intervention. In the present study, the mRNA expression of *IFITs* was found to be higher in LTBI cases compared to TB and healthy controls. Our results indicate the association of *IFITs* with the containment of mycobacteria in LTBI cases while failing to do the same in active-TB cases. It is noteworthy that we observed a trend of higher expression of *IFITs* in THP-1 cells infected with *M. smegmatis* followed by *M. bovis* BCG and *M. tb* R179, indicating that expression of *IFITs* may be affected by the virulence of mycobacterial species. A previous study showed that *IFITs* regulate interferon and other cytokine responses in LPS-activated human macrophages (John et al., 2018). Another study using *IFIT2-*deficient mice demonstrated that *IFIT2* is a critical signalling intermediate for LPS-induced septic shock. *IFIT2* expression was significantly upregulated in response to LPS challenge in an IFN-α receptor and IFN regulatory factor (Irf)9-dependent manner (Siegfried et al., 2013). Furthermore, another study showed that forced IFIT-2 expression represses LPS induced TNF-α expression at posttranscriptional levels (Berchtold et al., 2008). Thus, there is the likelihood that these factors play important role in thespectrum of *IFITs* expression which might help in the containment of the mycobacterial growth. This finding warrants more future studies into this area, which may pave the foundation for immunotherapeutic targets for TB.

## Limitations Of The Study

Limitations of the study are as follows: (a) luminex for the combined effect of *IFITs* overexpression and knock-down has not been done; (b) other than IFN-β, we have not explored other factors that may affect *IFITs*’ role in mycobacteria; and (c) the present study do not include function experiment and western blot. Also, the current manuscript is not a mechanistic study on the regulation of *IFIT*s; rather, we generated preliminary evidence that *IFITs* manipulation is associated with reduction of *in vitro* mycobacterial growth.

## Conclusion

Our finding suggests that *IFITs* play an important role during*in vitro*mycobacterial containment. Higher expression of *IFITs* in IFN-β-treated THP-1 cells prove that *IFITs* modulate the *in vitro* survival of mycobacteria by altering IFN-β levels. Overexpression/knock-down of *IFITs* inside macrophages causes a significant increase/decrease in key pro-inflammatory cytokines (IDO-1, IL-6, IL-23, and IFN-γ) resulting in the mycobacterial killing/survival, respectively. Higher expression of *IFITs*in LTBI cases compared to active TB cases indicates that *IFITs*, together with other host factors, control the mycobacterial survival. Differentially expressed *IFITs* showed a strong effect against mycobacteria, which can be used as a promising therapeutic target adjunct to anti-TB therapy. This knowledge will broaden the scope of host drug targets for resistance-free bacteriostatic immuno-therapy.

## Patent

The technology for using *IFITs* polypeptides for treating tuberculosis has been patented by our laboratory (Madhvi and Baker).

## Data Availability Statement

The de-identified patient data, study protocol, informed consent form, and datasets generated during and/or analysed during the study are available from the corresponding authors on reasonable request.

## Author Contributions

AM and BB designed the experiments. AM, HM performed the experiments. AM, HM, and NC analyzed the data. All authors wrote and edited the manuscript.

## Funding

This work is supported by the DSI-NRF Centre of Excellence for Biomedical Tuberculosis Research; South African Medical Research Council Centre for Tuberculosis Research; and Division of Molecular Biology and Human Genetics, Faculty of Medicine and Health Sciences, Stellenbosch University, Cape Town. Another support is from Harry Crossley Foundation, South Africa.

## Conflict of Interest

The authors declare that the research was conducted in the absence of any commercial or financial relationships that could be construed as a potential conflict of interest

## Publisher’s Note

All claims expressed in this article are solely those of the authors and do not necessarily represent those of their affiliated organizations, or those of the publisher, the editors and the reviewers. Any product that may be evaluated in this article, or claim that may be made by its manufacturer, is not guaranteed or endorsed by the publisher.
