# Supplementary material for: Increased Interferon-Induced Protein With Tetracopeptides (IFITs) Reduces Mycobacterial Growth
Source: Front Cell Infect Microbiol. 2022 Jul 5;12:828439. doi: 10.3389/fcimb.2022.828439 (PMC9296360; doi:10.3389/fcimb.2022.828439)
Supplement: Supplementary file 1 [file DataSheet_1.pdf]

Supplementary data

Table of Contents

Supplementary Figure 1: Flow of methodology adopted for the knock-up and knock-down experiments......... 2

Supplementary Figure 2: Flow diagram depicting the effect of co-stimulation with IFN-α, IFN-β and IFN-γ on expression of IFITs... 3

Supplementary Figure 3: Flow diagram describing knock-up and knock-down of IFITs.................................. 4

Supplementary Figure 5: Titration of IFITs for knock-up at different concentrations....................................... 6

Supplementary Figure 6: Viability of THP-1 cells after knock-down and knock-up of *IFITs*............................ 7

Supplementary Figure 7: Comparison of bacterial uptake by THP-1 cells across different mycobacterial species 8

Supplementary Figure 8a: Fold changes of different mRNA detected by qRT-PCR for clinical and *in vitro* protocol.. 9

Supplementary Figure 8b: Fold changes of different mRNA detected by qRT-PCR for clinical and *in vitro* protocol.. 10

Supplementary Figure 8c: Fold changes of different mRNA detected by qRT-PCR for clinical and *in vitro* protocol.. 11

Supplementary Table 1: Demographic and clinical information of study participants.................................... 12

Supplementary Table 2: Effect of in vitro knock-up and knock-down of IFITs on CFUs of M. smegmatis, M. bovis BCG and M. tb R179.. 13

Supplementary Table 3: Mycobacterial species and plasmids used for the *in vitro* knock-up and knock-down of *IFITs* in the study. 15

Supplementary Table 4: Cytokines expression (pg/ml) 12 hours post ***M. smegmatis*** infection†. Compared to *M. smegmatis* infected THP-1 cells without intervention, ... 16

Supplementary Table 5: Cytokine expression (pg/ml) after 12 and 96 hours of ***M. bovis* BCG** infection. Compared to BCG infected THP-1 cells without intervention 19

Supplementary Table 6: Cytokines expression (pg/ml) after 12 and 96 hours of ***M. tb* R179** infection of THP-1 cells.. 24

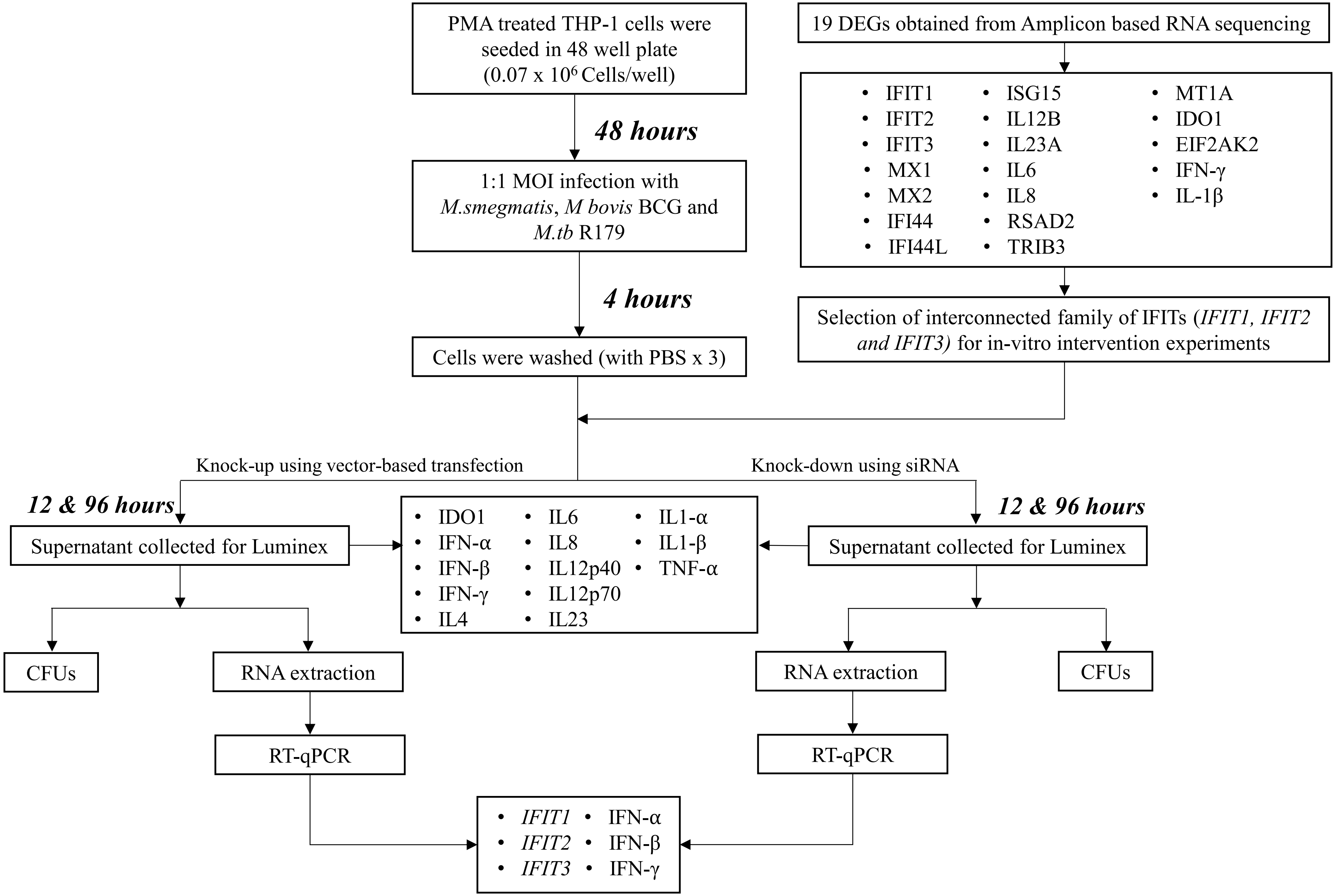

Supplementary Figure 1: Flow of methodology adopted for the knock-up and knock-down experiments.

Abbreviations: CFUs= colony forming units, BCG=Bacillus Calmette–Guerin, DEGs= Differentially expressed genes, EIF2AK2=eukaryotic translation initiation factor 2 alpha kinase 2, FDR=false discovery rate, IDO=indoleamine 2,3-dioxygenase, hMDMs=human monocyte-derived macrophages, IFI=interferon-induced protein, IFIT=interferon-induced protein with tetratricopeptide, IFN=interferon gamma, IL=interleukin, ISG=interferon-stimulated gene, MOI= multiplicity of infection, MTA=metastasis-associated protein, MX=interferon-induced GTP binding protein, PMA= Phorbol 12-myristate 13-acetate, RSAD=radical S-adenosyl methionine domain-containing protein 2, qRT-PCR= Quantitative reverse transcription polymerase chain reaction, TRIB= trible homolog, UBC=polyubiquitin-C.

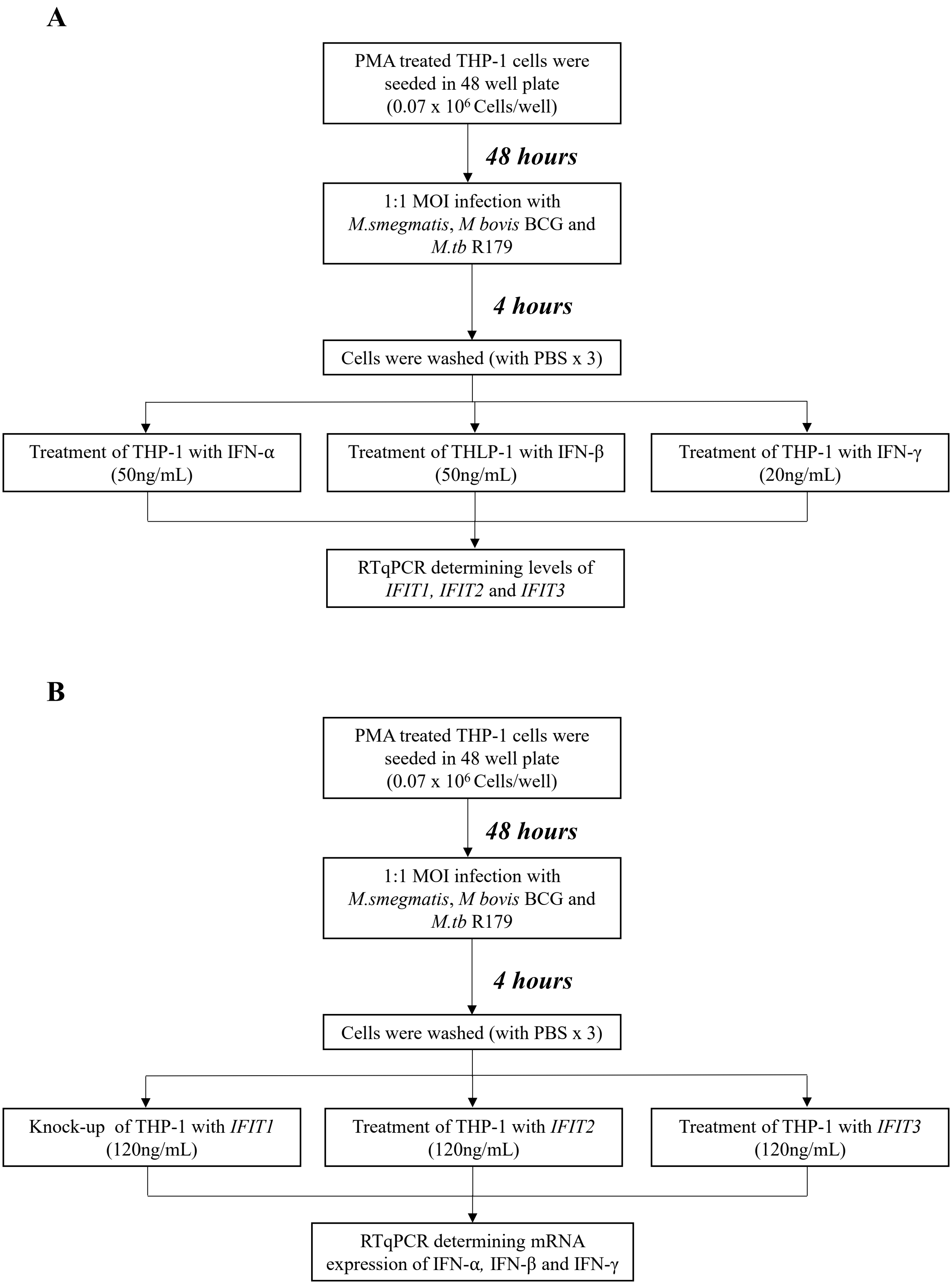

Supplementary Figure 2: Flow diagram depicting the effect of co-stimulation with IFN-α, IFN-β and IFN-γ on expression of IFITs determined by qRT-PCR (A) and the effect of knock-up of *IFITs* on the mRNA expression of IFN-α, IFN-β and IFN-γ (B).

Abbreviations: PMA= Phorbol Myristate Acetate, MOI= Multiplicity of infection, BCG= Bacillus Calmette-Guérin, PBS= Phosphate-buffered saline, THP-1 cells= human monocyte derived macrophages, IFN= Interferon, IFIT= Interferon-induced protein with tetratricopeptide repeats

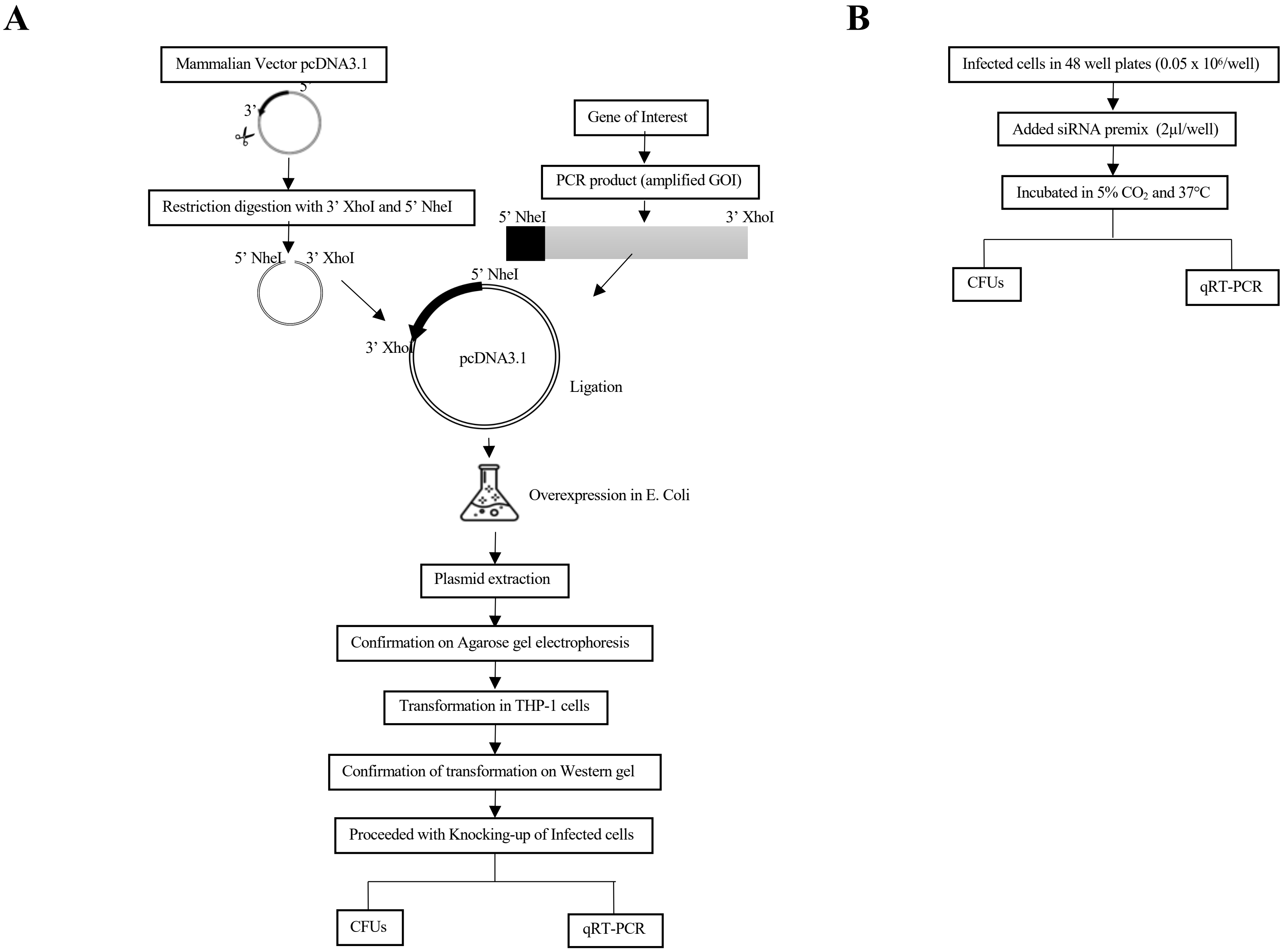

Supplementary Figure 3: Flow diagram describing knock-up and knock-down of IFITs. Flow depicts (A) cloning and vector (*E.* Coli) derived overexpression of *IFITs* (knock-up) and (B) siRNA targeting *IFITs* (knock-down) in THP-1 cells.

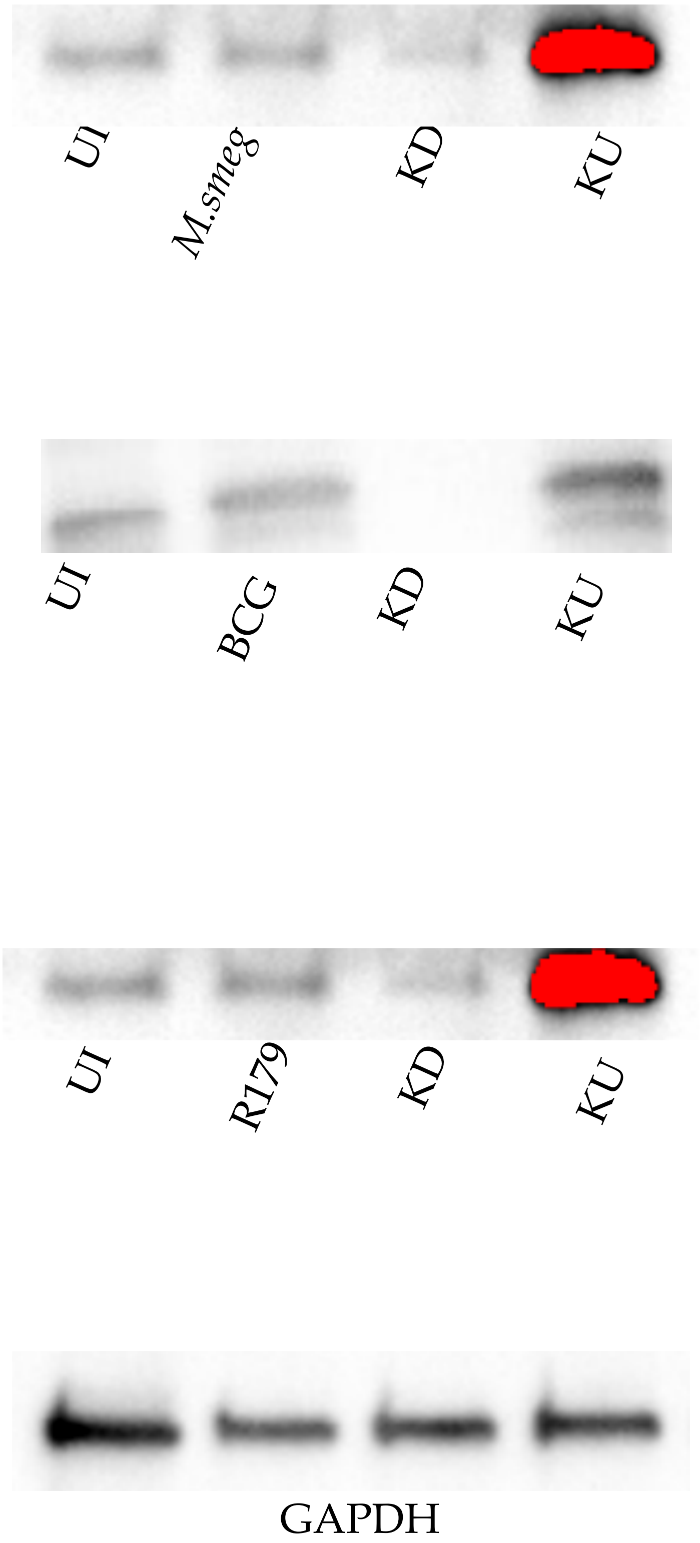

Supplementary Figure 4: Representative Western Blot confirming knock-down and knock-up (vector based overexpression) of IFIT1 in THP-1 infected with *M.smegmatis*, *M bovis BCG,* and *R179* at 12-hours post infection (*IFIT1-55KDa, GAPDH-37KDa)*

Abbreviations: KD=Knock-down, KU=Knock-up, UI=Uninfected

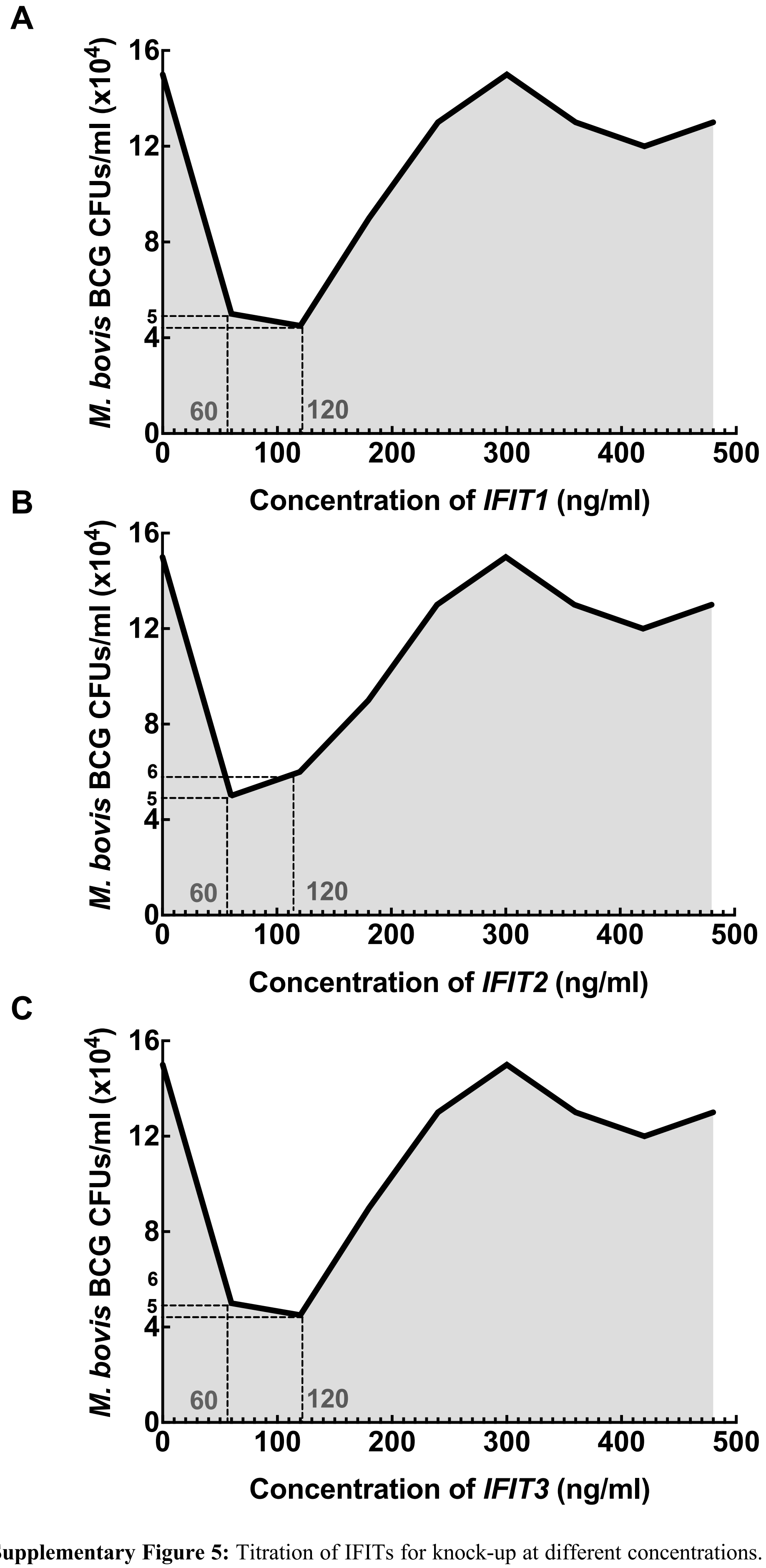

Supplementary Figure 5: Titration of IFITs for knock-up at different concentrations. Panel shows (A) *IFIT1,* (B) *IFIT2* and (C) *IFIT3* at different concentrations for knock-up in colony forming units of *M. bovis* BCG. We found concentration of 120 ng/ml to be most effective in reducing *in vitro* CFUs of BCG. *Abbreviation:* BCG= Bacillus Calmette–Guerin, IFIT= interferon-induced protein with tetratricopeptide.

A

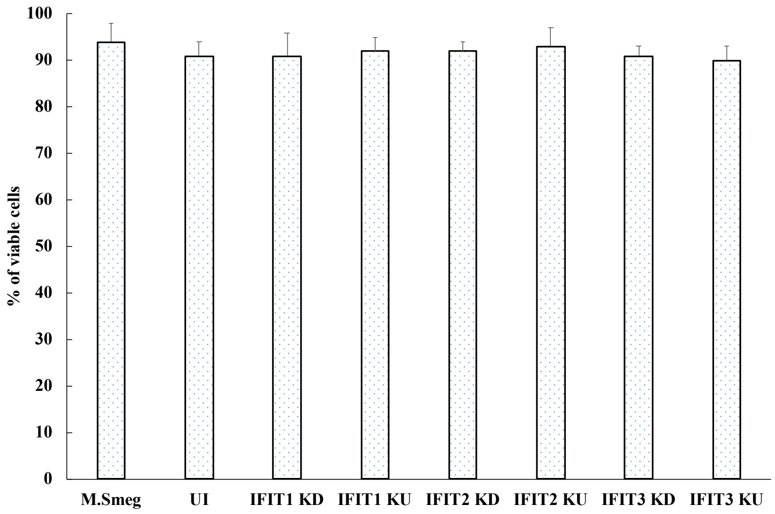

B

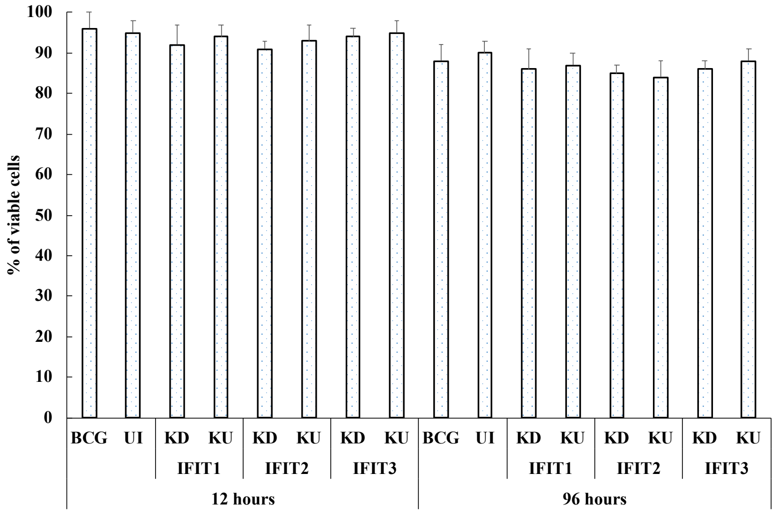

C

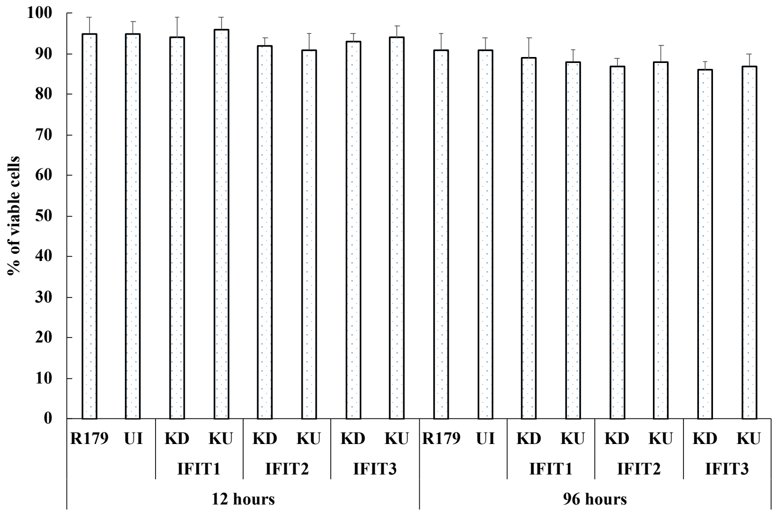

Supplementary Figure 6: Viability of THP-1 cells after knock-down and knock-up of *IFITs* infected with A) *M. smegmatis*, B) *M bovis* BCG and C) *M. tb* R179. The viability of the cells was found to be >85%, which was consistent and similar across the three species and knock-up and knock-down.
Abbreviations: KD= Knock-down, KU= Knock-up, UI= Uninfected

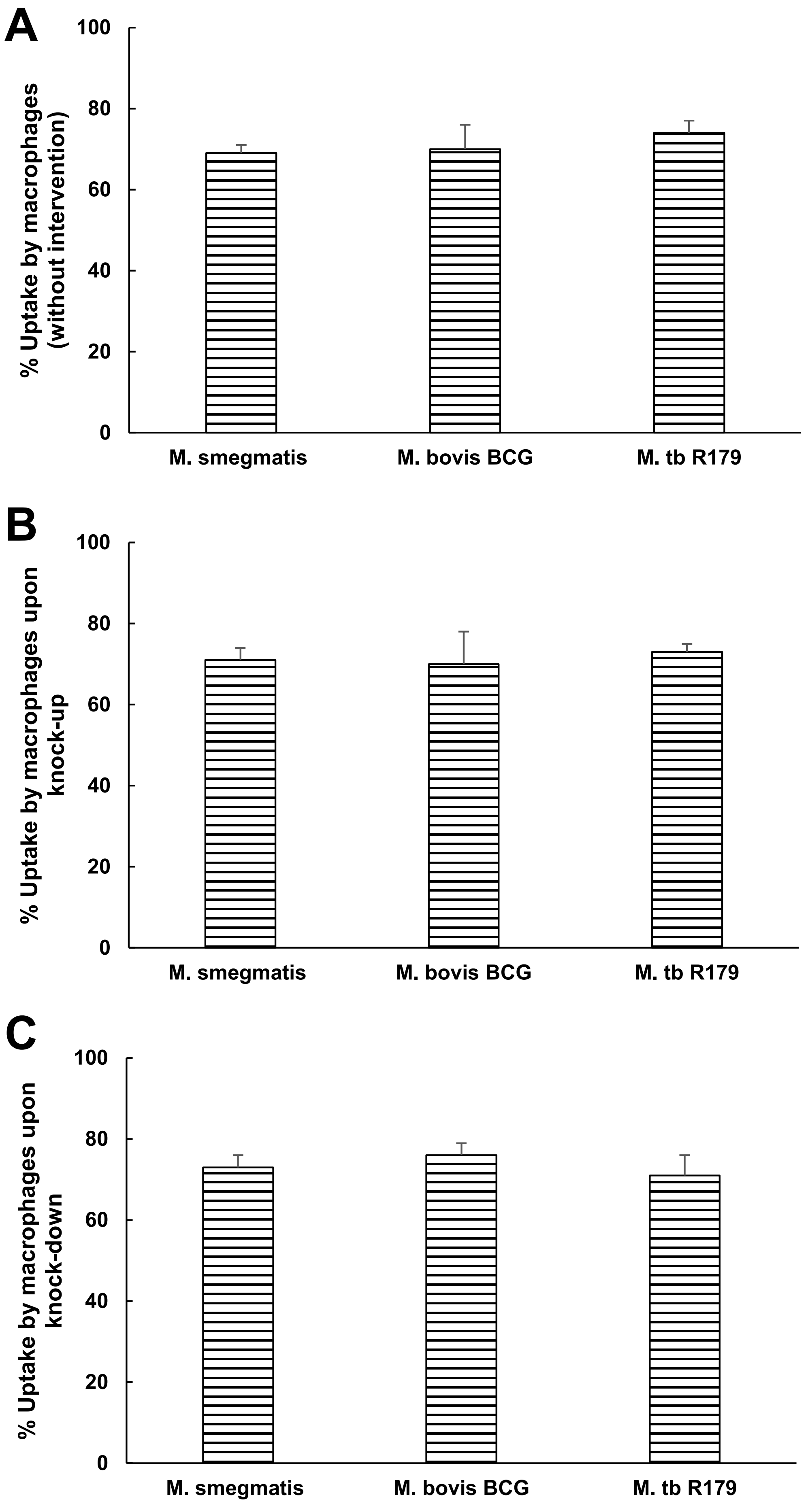

Supplementary Figure 7: Comparison of bacterial uptake by THP-1 cells across different mycobacterial species without intervention (A), after knock-up (B) and knock-down (C) of IFITs. The bacterial uptakes were found to be similar across different

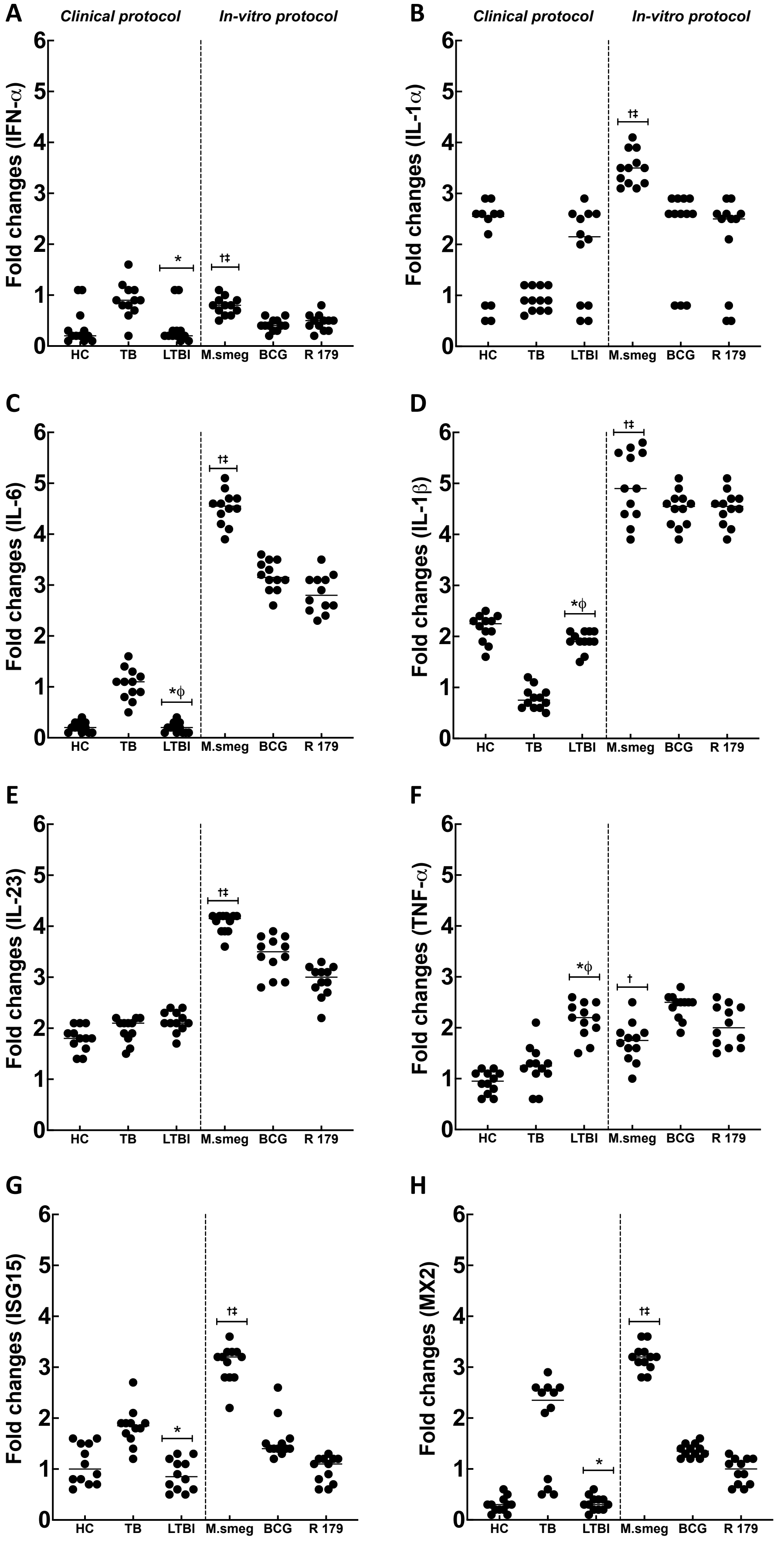

Supplementary Figure 8a: Fold changes of different mRNA detected by qRT-PCR for clinical and *in vitro* protocol. Compared to TB cases IFN-α, IL-6, ISG15 and MX2 were found to be lower, while TNF-α and IL-1β found to be higher in LTBI cases. For *in vitro* protocol, fold changes for *M. smegmatis* were found to be higher than *M. bovis* BCG and *M. tb* R179.
*= vs. TB, ϕ= vs. HC, †= vs. BCG, and ‡= vs. R179

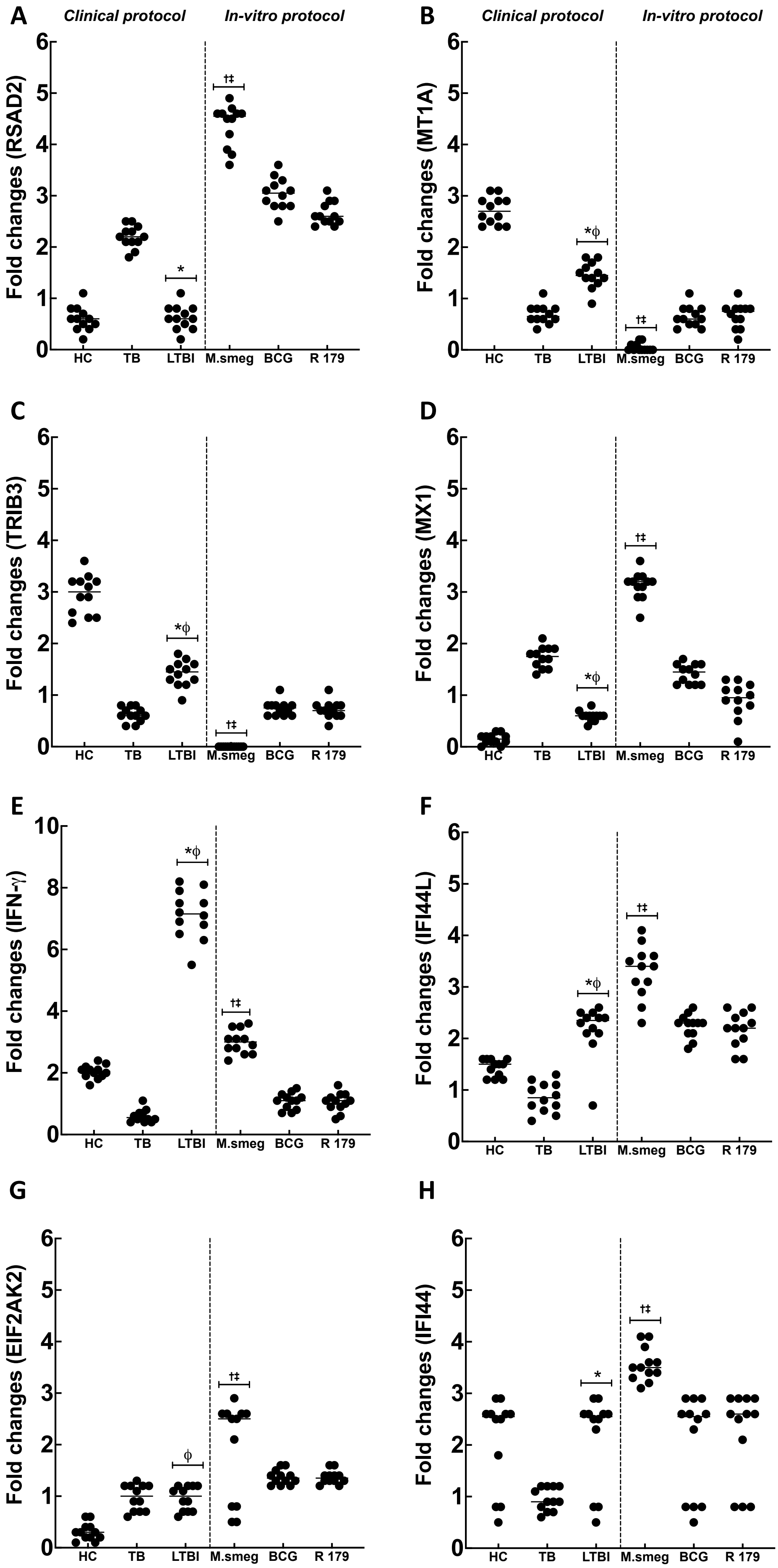

Supplementary Figure 8b: Fold changes of different mRNA detected by qRT-PCR for clinical and *in vitro* protocol. Compared to TB cases IFN-γ, TRIB3, MT1A, IFI44 and IFI44L were found to be lower, while RSAD2 and MX1 found to be higher in LTBI cases. For *in vitro* protocol, fold changes of RSAD2, MX1, IFN-γ, IFI44, IFI44L and EIF2AK2 were found to be higher, while TRIB3 and MT1A were lower for *M. smegmatis* compared to *M. bovis* BCG and *M. tb* R179.
*= vs. TB, ϕ= vs. HC, †= vs. BCG, and ‡= vs. R179

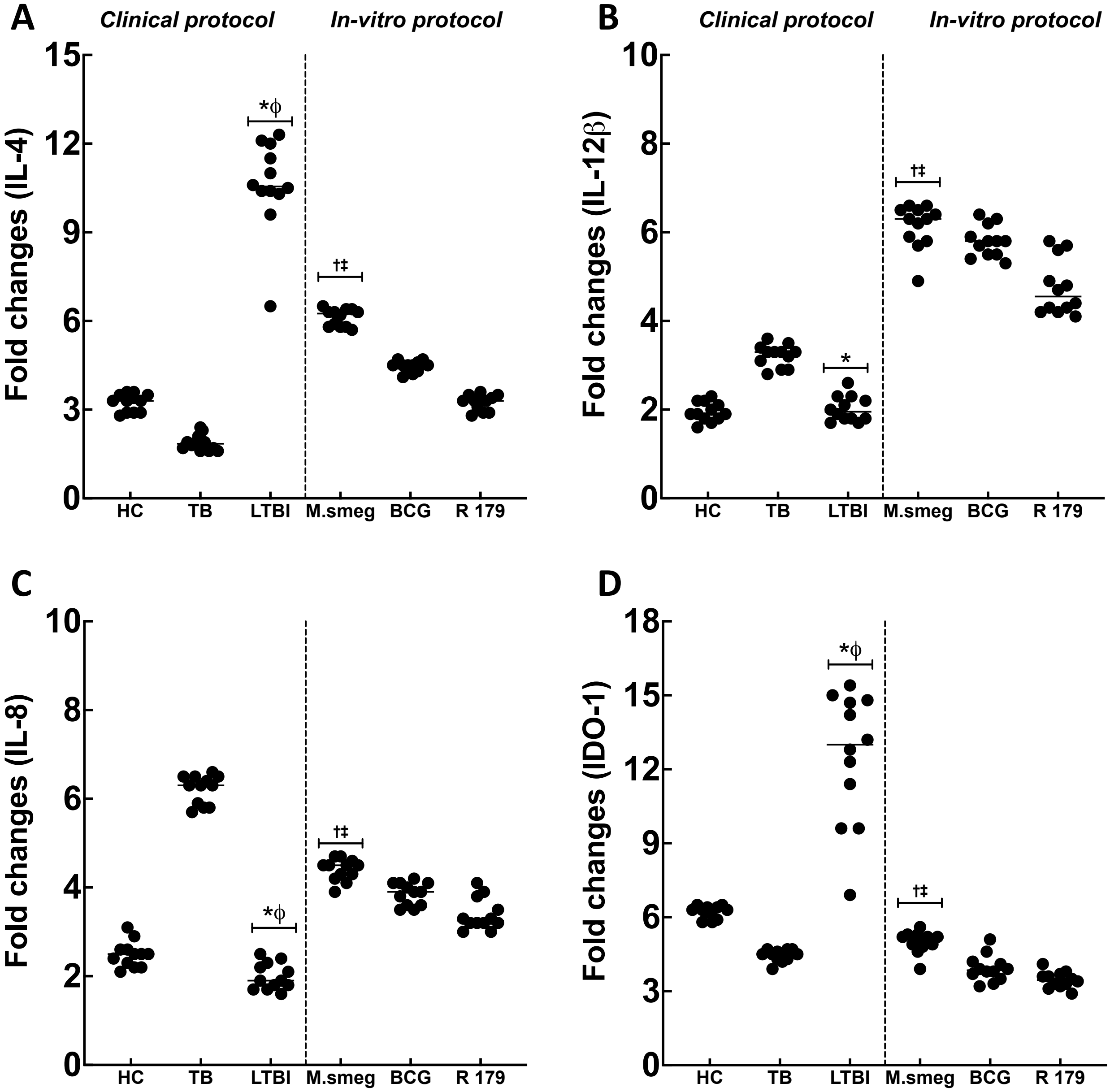

Supplementary Figure 8c: Fold changes of different mRNA detected by qRT-PCR for clinical and *in vitro* protocol. Compared to TB cases IL-4 and IDO-1 were found to be lower, while IL-8 and IL-12β found to be higher in LTBI cases. For *in vitro* protocol, fold changes were found to be higher in *M. smegmatis* compared to *M. bovis* BCG and *M. tb* R179.
*= vs. TB, ϕ= vs. HC, †= vs. BCG, and ‡= vs. R179

Supplementary Table 1: Demographic and clinical information of study participants. Data presented as mean ± S.D, median (IQR), and n/N (%).

	***In vitro* protocol**	**Clinical protocol (n=24)**	
	**Healthy controls (n=12)**	**LTBI cases (n=12)**	**Active TB cases (n=12)**
**Age (Years)**<br>Median (IQR)	27<br>(26-28.5)	37.2<br>(30.8-56.7)	49.5<br>(36.5-57)<br>p=0.406
**Gender (%)**			
**Female**	6/12 (50)	9/12 (75)	6/12 (33)
**Male**	6/12 (50)	3/12 (25)	6/12 (67)<br>p=0.206
**BMI (kg/m^2)**	23.59 ± 2.3	27.44 ± 9.1	26.36 ± 5.2<br>p=0.725

Abbreviations: BMI= body mass index, LTBI= latent tuberculosis infection, TB= Tuberculosis

Supplementary Table 2: Effect of in vitro knock-up and knock-down of IFITs on CFUs of M. smegmatis, M. bovis BCG and M. tb R179. Compared to untreated CFUs, we observed significant reduction in CFUs after knock-up of individual *IFIT1, IFIT2* and *IFIT3.* Also, higher CFUs observed after knock-down of individual *IFITs.* Combined effect of *IFITs* during knock-up and knock-down found to increase the survivability of *in vitro* CFUs. Results represented as increase (+%) or decrease (-%) in CFUs compared to untreated wells. Data represented as n and n/N (%).

	**Untreated CFUs**	**CFUs after Knock-up (x10^4)**				**CFUs after Knock-down (x10^4)**			
		IFIT1	***IFIT2***	***IFIT3***	***Average effect of knock-up***	***IFIT1***	***IFIT2***	***IFIT3***	***Average effect of knock-down***
M. smegmatis									
At 12 hours	41	30	29	26	-31%	85	89	90	+115%
% Change from untreated CFUs		-11/41 (-27%)	-12/41 (-29%)	-15/41 (-37%)		+44/41 (+107%)	+48/41 (+117%)	+49/41 (+120%)	
		**p<0.0001***	**p<0.0001***<br>p=0.806†	**p<0.0001***<br>p=0.343†<br>p=0.481‡		**p<0.0001***	**p<0.0001***<br>p=0.232†	**p<0.0001***<br>p=0.156†<br>p=0.812‡	
At 24 hours	15	8	7	6	-53%	26	26	28	+78%
% Change from untreated CFUs		-7/15 (-47%)	-8/15 (-53%)	-9/15 (-60%)		+11/15 (+73%)	+11/15 (+73%)	+13/15 (+87%)	
		**p=0.001***	**p=0.003***<br>p=0.715†	**p=0.006***<br>p=0.464†<br>p=0.713‡		**p=0.003***	**p=0.003***<br>p>0.999†	**p=0.002***<br>p=0.761†<br>p=0.761‡	
***M. bovis* BCG**									
At 12 hours	60	39	39	40	-34%	86	88	87	+45%
% Change from untreated CFUs		-21/60 (-35%)	-21/60 (-35%)	-20/60 (-33%)		+26/60 (+43%)	+28/60 (+47%)	+27/60 (+45%)	
		**p<0.0001***	**p<0.0001***<br>p>0.999†	**p<0.0001***<br>p=0.847†<br>p=0.847‡		**p<0.0001***	**p<0.0001***<br>p=0.821†	**p<0.0001***<br>p=0.909†<br>p=0.911‡	

Continue…

At 96 hours	71	49	49	51	-30%	97	101	102	+41%
% Change from untreated CFUs		-22/71<br>(-31%)	-22/71<br>(-31%)	-20/71<br>(-29%)		+26/71<br>(+37%)	+30/71<br>(+42%)	+31/71<br>(+44%)	
		**p<0.0001***	**p<0.0001***<br>p>0.999†	**p<0.0001***<br>p=0.713†<br>p=0.713‡		**p<0.0001***	**p<0.0001***<br>p=0.651†	**p<0.0001***<br>p=0.576†<br>p=0.915‡	
M. tb **R179**									
At 12 hours	51	39	39	38	-24%	84	89	89	+69%
% Change from untreated CFUs		-12/51<br>(-24%)	-12/51<br>(-24%)	-13/51<br>(-25%)		+33/51<br>(+65%)	+36/51<br>(+71%)	+36/51<br>(+71%)	
		**p<0.0001***	**p<0.0001***<br>p>0.999†	**p<0.0001***<br>p=0.818†<br>p=0.818‡		**p<0.0001***	**p<0.0001***<br>p=0.649†	**p<0.0001***<br>p=0.649†<br>p>0.999‡	
At 96 hours	74	43	42	41	-43%	99	100	99	+34%
% Change from untreated CFUs		-31/74<br>(-42%)	-32/74<br>(-43%)	-33/74<br>(-45%)		+25/74<br>(+34%)	+26/74<br>(+35%)	+25/74<br>(+34%)	
		**p<0.0001***	**p<0.0001***<br>p=0.868†	**p<0.0001***<br>p=0.740†<br>p=0.868‡		**p<0.0001***	**p<0.0001***<br>p=0.904†	**p<0.0001***<br>p<0.999†<br>p=0.904‡	
Overall (across all three species)		-32.5%<br>(-30% to 43%)				+57%<br>(+41% to +78%)			

*=vs. Untreated CFUs, †=vs. *IFIT1,* ‡=vs. *IFIT2,* $^{\$}$=vs. *IFIT3*

Abbreviations: BCG= Bacillus Calmette–Guérin, CFUs= colony forming units, *IFITs*= interferon-induced protein with tetratricopeptide repeats

Supplementary Table 3: Mycobacterial species and plasmids used for the *in vitro* knock-up and knock-down of *IFITs* in the study.

	**Description**	**Source/Reference**
M. tb species		
M. smegmatis	*M. smegmatis* MC155	Laboratory collection (Harper *et al*., 2010)
M. bovis BCG	*M. bovis* BCG strain Pasteur 1743P2	Laboratory collection (Viljoen *et al*., 2013)
M. tb R179	Beijing genotype strain R220	Clinical isolate (Johnson *et al*., 2006)
E. coli DH5α	ATCC53868	Laboratory collection
Plasmid for overexpression (knock-up)		
pcDNA3.1 3xFlag *IFIT1*	Mammalian expression vector with human *IFIT1* as an insert and without mutations	(Katibah *et al*., 2013)
pcDNA3.1 3xFlag *IFIT2*	Mammalian expression vector with human *IFIT2* as an insert without mutations	(Katibah *et al*., 2013)
pcDNA3.1 3xFlag *IFIT3*	Mammalian expression vector with human *IFIT3* as an insert without mutations	(Katibah *et al*., 2013)
***siRNA pre-mix of IFITs* (*knock-down*)**		
**siRNA Premix**	**Target Sequence (5' to 3')**	**Catalogue Number (length)**
Hs-IFIT1	CAGGCTGTCCGCTTAAATCCA	Catalogue no. S100445879 (4396 bp)
Hs-IFIT1	TACATGGGAGTTATCCATTGA	Catalogue no. S103224284 (4396 bp)
Hs-IFIT2	AAAGAAAGTTACTGGAACTAA	Catalogue no. S104145372 (3505 bp)
Hs-IFIT2	CCCATAGAGGTTAGTCCTGCA	Catalogue no. S104259010 (3505 bp)
Hs-IFIT3	ATGCTATGGACTATTCGAATA	Catalogue no. S103152737 (2467 bp)
Hs-IFIT3	AGAGATGATTGAAGCACTAAA	Catalogue no. S104197788 (2467 bp)

Abbreviations: BCG= Bacillus Calmette–Guérin, E. coli= Escherichia coli, *IFIT*= interferon-induced protein with tetratricopeptide.

Supplementary Table 4: Cytokines expression (pg/ml) 12 hours post ***M. smegmatis*** infection†. Compared to *M. smegmatis* infected THP-1 cells without intervention, we observed significantly lower level of cytokines after knock-down and higher level after knock-up of *IFITs*. For knock-down, levels of IFN-α, IFN-β, IL-1α, IL-4 and IL-12p70 were found to be similar. Also, for knock-up, levels of IL-1α, IL-1β and TNF-α were found to be similar. Data presented as mean ± S.D.

**Cytokines**	**Uninfected**	***M. smegmatis***	**Knock-down (siRNA)**			**Knock-up (vector-based over-expression)**		
			IFIT1	***IFIT2***	***IFIT3***	***IFIT1***	***IFIT2***	***IFIT3***
at 12 hours								
**IDO-1**	971 ± 13.4	1550.3 ± 16.4<br>**p =0.047**[*]	0.3 ± 0.5<br>**p<0.001**[*]<br>**p<0.001**[†]	8.6 ± 1.5<br>**p<0.001**[*]<br>**p<0.001**[†]	9 ± 1<br>**p<0.001**[*]<br>**p<0.001**[†]	1864.3 ± 5.8<br>**p=0.001**[*]<br>p=0.708[†]	2343 ± 7.5<br>**p<0.001**[*]<br>**p=0.001**[†]	2865 ± 9.8<br>**p<0.001**[*]<br>**p<0.001**[†]
**IFN-α**	29 ± 1	82 ± 6.2<br>p>0.999 [*]	0.3 ± 0.5<br>p>0.999 [*]<br>p>0.999[†]	6 ± 1<br>p>0.999 [*]<br>p>0.999[†]	12.5 ± 2<br>p>0.999 [*]<br>p>0.999[†]	1085 ± 9.8<br>**p=0.001**[*]<br>**p=0.003**[†]	1255.6 ± 9.4<br>**p=0.044**[*]<br>**p=0.001**[†]	984.6 ± 15.6<br>**p<0.001**[*]<br>**p<0.001**[†]
**IFN-β**	0	0<br>p>0.999[*]	0<br>p>0.999[*]<br>p>0.999[†]	0<br>p>0.999[*]<br>p>0.999[†]	0<br>p>0.999[*]<br>p>0.999[†]	635 ± 12.5<br>**p<0.001**[*]<br>**p<0.001**[†]	414.6 ± 8.5<br>**p<0.001**[*]<br>**p<0.001**[†]	488.3 ± 8<br>**p<0.001**[*]<br>**p<0.001**[†]
**IFN-γ**	527.3 ± 12	1361 ± 7.5<br>**p=0.0004**[*]	0<br>p=0.101[*]<br>**p<0.001**[†]	0<br>p=0.101[*]<br>**p<0.001**[†]	0<br>p=0.101[*]<br>**p<0.001**[†]	7599 ± 946.8<br>**p<0.001**[*]<br>**p<0.001**[†]	7405.3 ± 1029.2<br>**p<0.001**[*]<br>**p<0.001**[†]	7670 ± 908.8<br>**p<0.001**[*]<br>**p<0.001**[†]
**IL-4**	7.6 ± 4.7	487.3 ± 9.6<br>**p<0.001**[*]	0<br>p>0.999[*]<br>**p=0.016**[†]	0.3 ± 0.5<br>p>0.999[*]<br>**p=0.017**[†]	2.3 ± 2.5<br>p>0.999[*]<br>**p=0.017**[†]	5164.3 ± 179.8<br>**p<0.001**[*]<br>**p<0.001**[†]	5134.3 ± 305.1<br>**p<0.001**[*]<br>**p<0.001**[†]	4954.6 ± 492.2<br>**p<0.001**[*]<br>**p<0.001**[†]

[*]vs. Uninfected, [†]vs. *M. smegmatis*

**IL-6**	239.3 ± 19.8	1983.6 ± 4<br>**p<0.001***	0.6 ± 0.5<br>p=909 *<br>**p<0.001** †	9 ± 8.1<br>p=0.924 *<br>**p<0.001** †	0.6 ± 1.1<br>p=0.909 *<br>**p<0.001** †	3530.6 ± 467.3<br>**p<0.001***<br>**p<0.001** †	3567.3 ± 216.7<br>**p<0.001***<br>**p<0.001** †	2921 ± 84.1<br>**p<0.001***<br>**p<0.001** †
**IL-8**	657 ± 19.6	9442.6 ± 489.8<br>**p<0.001** *	1300.6 ± 3<br>**p= 0.016***<br>**p<0.002**†	676.3 ± 19.8<br>p=0.999 *<br>**p=0.001** †	991.6 ± 38.2<br>p=0.635 *<br>**p<0.001** †	8680.6 ± 84.5<br>**p<0.001***<br>**p= 0.002**†	8932.6 ± 52.5<br>**p<0.001***<br>p=0.125 †	7451.6 ± 797.8<br>**p<0.001***<br>**p<0.001** †
**IL-12p40**	94.6 ± 6.6	753.6 ± 9.7<br>p=0.013 *	3.6 ± 3.2<br>p=0.999 *<br>**p= 0.002**†	9.3 ± 4.5<br>p=0.924 *<br>**p=0.003** †	0<br>p=0.999 *<br>**p=0.002**†	3421.6 ± 477.3<br>**p<0.001***<br>**p<0.001** †	2591 ± 360.2<br>**p<0.001***<br>**p<0.001** †	3828 ± 164.8<br>**p<0.001***<br>**p<0.001** †
**IL-12p70**	16.6 ± 4.1	406.3 ± 14.7<br>p=0.437 *	0.3 ± 0.5<br>p>0.999 *<br>**p=0.038** †	0<br>p>0.999 *<br>**p=0.038** †	0<br>p>0.999 *<br>**p=0.038** †	561.6 ± 8.7<br>p=0.078 *<br>p= 0.991†	844.6 ± 32<br>**p=0.001** *<br>p= 0.283†	767.6 ± 32.6<br>**p=0.002** *<br>p=0.539 †
**IL-23**	1245 ± 52.7	2069.6 ± 122.9<br>**p=0.001** *	0.6 ± 1.1<br>**p<0.001** *<br>**p<0.001** †	0<br>**p<0.001** *<br>**p<0.001** †	2 ± 3.4<br>**p<0.001** *<br>**p<0.001** †	4599.3 ± 510.1<br>**p<0.001** *<br>**p<0.001** †	4547.3 ± 253.6<br>**p<0.001** *<br>**p<0.001** †	3207.3 ± 179.4<br>**p<0.001** *<br>**p<0.001** †

*vs. Uninfected, †vs. *M. smegmatis*

**IL-1α**	243.3 ± 9.4	254.3 ± 40.5<br>p<0.999 *	67.3 ± 3<br>p= 0.982*<br>p= 0.975†	74.6 ± 10.9<br>p=0.986 *<br>p= 0.980†	75 ± 8.7<br>p=0.986 *<br>p= 0.980†	6932.3 ± 79<br>**p<0.001** *<br>**p<0.001** †	5453 ± 380.7<br>**p<0.001** *<br>**p<0.001** †	4562.6 ± 312.5<br>**p<0.001** *<br>**p<0.001** †
**IL-1β**	22 ± 4.3	131 ± 13.1<br>**p=0.0002** *	8.6 ± 3.2<br>p=0.982 *<br>p=0.998 †	7 ± 2.6<br>p=0.986 *<br>p=0.998 †	1 ± 1<br>p=0.986 *<br>p=0.997 †	1278 ± 151.6<br>**p<0.001** *<br>**p<0.001** †	1484.6 ± 41.7<br>**p<0.001** *<br>**p<0.001** †	1160.6 ± 165.7<br>**p<0.001** *<br>**p<0.001** †
**TNF-α**	30 ± 5.5	634.3 ± 33.8<br>p=0.032 *	139.3 ± 23.1<br>p= 0.999*<br>p= 0.151†	264.6 ± 20.6<br>p= 0.916*<br>p=0.508 †	113.6 ± 9.5<br>p<0.999 *<br>p= 0.109†	4456.6 ± 318.1<br>**p<0.001** *<br>**p<0.001** †	6202 ± 168.3<br>**p<0.001** *<br>**p<0.001** †	4154.6 ± 123.1<br>**p<0.001** *<br>**p<0.001** †

*vs. Uninfected, †vs. *M. smegmatis*

Abbreviations: IDO, indoleamine 2,3-dioxygenase; IFI, interferon-induced protein; IFIT, interferon-induced protein with tetratricopeptide; IFN, interferon gamma; IL, interleukin; ISG, interferon-stimulated gene

Supplementary Table 5: Cytokine expression (pg/ml) after 12 and 96 hours of ***M. bovis*** **BCG** infection. Compared to BCG infected THP-1 cells without intervention, we observed higher levels of cytokines for knock-down and knock-up of *IFITs*. The level of most of the cytokines increases significantly after knock-up of *IFITs*. For knock-down, levels of IFN-α, IFN-β, IL-4 and IL-12p70 were found to be similar. Also, for knock-up, levels of IL-1α, IL-1β and TNF-α were found to be similar. Data presented as mean ± S.D

Cytokines	Uninfected	*M. bovis* BCG	Knock-down (siRNA)			Knock-up (vector-based over-expression)		
			IFIT1	*IFIT2*	*IFIT3*	*IFIT1*	*IFIT2*	*IFIT3*
at 12 hours								
**IDO-1**	971 ± 13.4	1489.3 ± 112.2<br>p =0.073*	0.3 ± 0.5<br>**p<0.001***<br>**p<0.001†**	8.6 ± 1.5<br>**p<0.001***<br>**p<0.001†**	9 ± 1<br>**p<0.001***<br>**p<0.001†**	3201.3 ± 86.6<br>**p<0.001***<br>**p<0.001†**	3198 ± 171.4<br>**p<0.001***<br>**p<0.001†**	4069.3 ± 141.9<br>**p<0.001***<br>**p<0.001†**
**IFN-α**	29 ± 1	83.6 ± 9<br>p>0.999 *	0.3 ± 0.5<br>p>0.999 *<br>p>0.999†	6 ± 1<br>p>0.999 *<br>p>0.999†	12.6 ± 2<br>p>0.999 *<br>p>0.999†	769.6 ± 28.5<br>**p=0.001***<br>**p=0.003†**	579.6 ± 36.1<br>**p=0.044***<br>p=0.101†	872 ± 103.7<br>**p<0.001***<br>**p=0.0004†**
**IFN-β**	0	0<br>p>0.999*	0<br>p>0.999*<br>p>0.999†	0<br>p>0.999*<br>p>0.999†	0<br>p>0.999*<br>p>0.999†	8225.6 ± 413.2<br>**p<0.001***<br>**p<0.001†**	5366.3 ± 304<br>**p<0.001***<br>**p<0.001†**	6498.6 ± 160.9<br>**p<0.001***<br>**p<0.001†**
**IFN-γ**	527.3 ± 12	1691 ± 84.7<br>**p<0.001***	0<br>p=0.064*<br>**p<0.001†**	0<br>p=0.064*<br>**p<0.001†**	0<br>p=0.064*<br>**p<0.001†**	6937.3 ± 369.8<br>**p<0.001***<br>**p<0.001†**	8228.3 ± 404.2<br>**p<0.001***<br>**p<0.001†**	6273.3 ± 370.5<br>**p<0.001***<br>**p<0.001†**
**IL-4**	7.6 ± 4.7	829 ± 31.9<br>**p=0.0002***	0<br>p>0.999*<br>**p<0.001†**	0.3 ± 0.5<br>p>0.999*<br>**p<0.001†**	2.3 ± 2.5<br>p>0.999*<br>**p<0.001†**	5797.6 ± 810.4<br>**p<0.001***<br>**p<0.001†**	5019.6 ± 290.5<br>**p<0.001***<br>**p<0.001†**	4559.6 ± 252.1<br>**p<0.001***<br>**p<0.001†**

*vs. Uninfected, †vs. *M. bovis* BCG

**IL-6**	239.3 ± 19.8	2376 ± 319<br>**p<0.001**[*]	0.6 ± 0.5<br>p=0.880[*]<br>**p<0.001**[†]	9 ± 8.1<br>p=0.898[*]<br>**p<0.001**[†]	0.6 ± 1.1<br>p=0.880[*]<br>**p<0.001**[†]	9253.3 ± 378.3<br>**p<0.001**[*]<br>**p<0.001**[†]	6491 ± 157.4<br>**p<0.001**[*]<br>**p<0.001**[†]	8353.6 ± 225.3<br>**p<0.001**[*]<br>**p<0.001**[†]
**IL-8**	657 ± 19.6	9113 ± 572<br>**p<0.001**[*]	1854.6 ± 127.1<br>**p<0.001** [*]<br>**p<0.001**[†]	1106.6 ± 72.5<br>p=0.186 [*]<br>**p<0.001**[†]	1799.3 ± 56.5<br>**p<0.001** [*]<br>**p<0.001**[†]	8328 ± 616.3<br>**p<0.001** [*]<br>**p<0.001**[†]	8526 ± 677.8<br>**p<0.001** [*]<br>**p=0.024**[†]	7928 ± 54.5<br>**p<0.001** [*]<br>**p<0.001**[†]
**IL-12p40**	94.6 ± 6.6	493.6 ± 60<br>p=0.327[*]	3.6 ± 3.2<br>p<0.999[*]<br>p=0.110[†]	9.3 ± 4.5<br>p<0.999[*]<br>p=0.110[†]	0<br>p<0.999[*]<br>p=0.110[†]	3241.3 ± 652.6<br>**p<0.001**[*]<br>**p<0.001**[†]	2480 ± 419.2<br>**p<0.001**[*]<br>**p<0.001**[†]	1080.6 ± 61.2<br>**p<0.001**[*]<br>p=0.024[†]
**IL-12p70**	16.6 ± 4.1	375.3 ± 59.9<br>p=0.469[*]	0.3 ± 0.5<br>p<0.999[*]<br>p=0.409[†]	0<br>p<0.999[*]<br>p=0.408[†]	0<br>p<0.999[*]<br>p=0.408[†]	1538.6 ± 26.6<br>**p<0.001**[*]<br>**p<0.001**[†]	1087 ± 17.6<br>**p<0.001**[*]<br>**p<0.001**[†]	1968 ± 195<br>**p<0.001**[*]<br>**p<0.001**[†]
**IL-23**	1245 ± 52.7	2205 ± 213.6<br>**p<0.001**[*]	528 ± 102.8<br>**p=0.002**[*]<br>**p<0.001**[†]	147.3 ± 25.5<br>**p<0.001**[*]<br>**p<0.001**[†]	376.3 ± 39.4<br>**p<0.001**[*]<br>**p<0.001**[†]	6530 ± 453.2<br>**p<0.001**[*]<br>**p<0.001**[†]	6558 ± 258.9<br>**p<0.001**[*]<br>**p<0.001**[†]	4408.6 ± 495.2<br>**p<0.001**[*]<br>**p<0.001**[†]
**IL-1α**	243.3 ± 9.4	254.6 ± 40.5<br>p>0.999[*]	72.6 ± 11.7<br>p=0.979[*]<br>p=0.970[†]	67.6 ± 21.1<br>p=0.974[*]<br>p=0.965[†]	57.3 ± 26.3<br>p=0.966[*]<br>p=0.953[†]	786.3 ± 71.2<br>p=0.050[*]<br>p=0.060[†]	621.6 ± 95<br>p=0.390[*]<br>p=0.438[†]	1139.6 ± 122.5<br>**p<0.001**[*]<br>**p<0.001**[†]
**IL-1β**	22 ± 4.3	127 ± 15.6<br>p=0.998[*]	12.3 ± 8.7<br>p>0.999[*]<br>p=0.998[†]	12 ± 7.2<br>p>0.999[*]<br>p=0.998[†]	2.3 ± 1.5<br>p>0.999[*]<br>p=0.996[†]	752.3 ± 33.2<br>**p=0.001**[*]<br>**p=0.012**[†]	743.3 ± 26.8<br>**p=0.002**[*]<br>**p=0.014**[†]	597.3 ± 65.2<br>**p=0.029**[*]<br>p=0.144[†]
**TNF-α**	30 ± 5.5	679.3 ± 103.3<br>**p=0.007**[*]	104 ± 11<br>p>0.999[*]<br>**p=0.029**[†]	56.3 ± 16.6<br>p>0.999[*]<br>**p=0.012**[†]	14 ± 2<br>p>0.999[*]<br>**p=0.005**[†]	8295 ± 303.9<br>**p<0.001**[*]<br>**p<0.001**[†]	8200.6 ± 608.7<br>**p<0.001**[*]<br>**p<0.001**[†]	6406 ± 168<br>**p<0.001**[*]<br>**p<0.001**[†]

[*]vs. Uninfected, [†]vs. *M. bovis* BCG

**Cytokines**	**Uninfected**	***M. bovis* BCG**	**Knock-down (siRNA)**			**Knock-up (vector-based over-expression)**		
			IFIT1	***IFIT2***	***IFIT3***	***IFIT1***	***IFIT2***	***IFIT3***
at 96 hours								
**IDO-1**	971 ± 13.4	1504.3 ± 86.5<br>p=0.112[*]	0.3 ± 0.5<br>**p<0.001**[*]<br>**p<0.001**[†]	8.6 ± 1.5<br>**p<0.001**[*]<br>**p<0.001**[†]	9 ± 1<br>**p<0.001**[*]<br>**p<0.001**[†]	2525.3 ± 346.7<br>**p<0.001**[*]<br>**p<0.001**[†]	1236.6 ± 185.1<br>p=0.869[*]<br>p=0.864[†]	2724 ± 249<br>**p<0.001**[*]<br>**p<0.001**[†]
**IFN-α**	29 ± 1	74 ± 7.9<br>p>0.999 [*]	0.3 ± 0.5<br>p>0.999 [*]<br>p>0.999 [†]	6 ± 1<br>p>0.999[*]<br>p>0.999[†]	12.6 ± 2<br>p>0.999[*]<br>p>0.999[†]	534 ± 74<br>p=0.158[*]<br>p=0.258[†]	131.6 ± 9<br>p=0.995[*]<br>p>0.999 [†]	664.6 ± 34<br>**p=0.026**[*]<br>p=0.052[†]
**IFN-β**	0	0<br>p>0.999[*]	0<br>p>0.999 [*]<br>p>0.999 [†]	0<br>p>0.999[*]<br>p>0.999[†]	0<br>p>0.999[*]<br>p>0.999[†]	6310.3 ± 248.4<br>**p<0.001**[*]<br>**p<0.001**[†]	5512.6 ± 101<br>**p<0.001**[*]<br>**p<0.001**[†]	4534 ± 365.8<br>**p<0.001**[*]<br>**p<0.001**[†]
**IFN-γ**	527.3 ± 12	964.3 ± 30<br>p=0.322[*]	0<br>p=0.121[*]<br>**p<0.001**[†]	0<br>p=0.121[*]<br>**p<0.001**[†]	0<br>p=0.121[*]<br>**p<0.001**[†]	5936.3 ± 635.3<br>**p<0.001**[*]<br>**p<0.001**[†]	8237.3 ± 459.7<br>**p<0.001**[*]<br>**p<0.001**[†]	7457.6 ± 298.2<br>**p<0.001**[*]<br>**p<0.001**[†]
**IL-4**	7.6 ± 4.7	492 ± 13.4<br>p=0.200[*]	0<br>p>0.999 [*]<br>p=0.184[†]	0.3 ± 0.5<br>p>0.999[*]<br>p=0.184[†]	2.3 ± 2.5<br>p>0.999[*]<br>p=0.188[†]	4410.3 ± 177.9<br>**p<0.001**[*]<br>**p<0.001**[†]	4216 ± 102.8<br>**p<0.001**[*]<br>**p<0.001**[†]	3453.6 ± 366.5<br>**p<0.001**[*]<br>**p<0.001**[†]

[*]vs. Uninfected, [†]vs. *M. bovis* BCG

**IL-6**	239.3 ± 19.8	2347.6 ± 315.4<br>**p<0.001**[*]	0.6 ± 0.5<br>p=0.921[*]<br>**p<0.001**[†]	9 ± 8.1<br>p=0.934[*]<br>**p<0.001**[†]	0.6 ± 1.1<br>p=0.921[*]<br>**p<0.001**[†]	6586 ± 492.9<br>**p<0.001**[*]<br>**p<0.001**[†]	7373.3 ± 163.5<br>**p<0.001**[*]<br>**p<0.001**[†]	4651 ± 385.9<br>**p<0.001**[*]<br>**p<0.001**[†]
**IL-8**	657 ± 19.6	9296.6 ± 445.6<br>**p<0.001**[*]	1208.3 ± 160.5<br>p=0.089[*]<br>**p<0.001**[†]	1479.3 ± 153.8<br>**p<0.001**[*]<br>**p<0.001**[†]	979.6 ± 20.9<br>p=0.708[*]<br>**p<0.001**[†]	3270.6 ± 1025.6<br>**p<0.001**[*]<br>**p<0.001**[†]	2677 ± 417.2<br>**p<0.001**[*]<br>**p<0.001**[†]	2662.3 ± 263.8<br>**p<0.001**[*]<br>**p<0.001**[†]
**IL-12p40**	94.6 ± 6.6	589.6 ± 59<br>p=0.178[*]	3.6 ± 3.2<br>p>0.999 [*]<br>p=0.055[†]	9.3 ± 4.5<br>p>0.999[*]<br>p=0.059[†]	0<br>p>0.999[*]<br>p=0.052[†]	1355 ± 527.6<br>**p<0.001**[*]<br>**p=0.003**[†]	2553.3 ± 370.2<br>**p<0.001**[*]<br>**p<0.001**[†]	2358.3 ± 433.3<br>**p<0.001**[*]<br>**p<0.001**[†]
**IL-12p70**	16.6 ± 4.1	23.6 ± 1.1<br>p>0.999[*]	0<br>p>0.999 [*]<br>p>0.999[†]	0.3 ± 0.5<br>p>0.999[*]<br>p>0.999[†]	0<br>p>0.999[*]<br>p>0.999[†]	1448 ± 264.7<br>**p<0.001**[*]<br>**p<0.001**[†]	1873.6 ± 126.1<br>**p<0.001**[*]<br>**p<0.001**[†]	2923 ± 346.1<br>**p<0.001**[*]<br>**p<0.001**[†]
**IL-23**	1245 ± 52.7	1077.6 ± 49.6<br>p=0.989[*]	407.3 ± 12<br>**p=0.001**[*]<br>**p=0.015**[†]	410.3 ± 13.5<br>**p=0.001**[*]<br>**p=0.016**[†]	231.6 ± 46.1<br>**p<0.001**[*]<br>**p=0.001**[†]	5143 ± 409.8<br>**p<0.001**[*]<br>**p<0.001**[†]	4478 ± 251.8<br>**p<0.001**[*]<br>**p<0.001**[†]	4507 ± 319.5<br>**p<0.001**[*]<br>**p<0.001**[†]
**IL-1α**	243.3 ± 9.4	262.6 ± 42.8<br>p>0.999[*]	14.6 ± 3<br>p=0.936[*]<br>p=0.904[†]	14.3 ± 3<br>p=0.936[*]<br>p=0.904[†]	38.3 ± 3<br>p=0.964[*]<br>p=0.942[†]	732.6 ± 112.4<br>p=0.189[*]<br>p=0.233[†]	551.6 ± 112.3<br>p=0.754[*]<br>p=0.810[†]	469.3 ± 45.4<br>p=0.940[*]<br>p=0.963[†]

[*]vs. Uninfected, [†]vs. *M. bovis* BCG

**IL-1β**	22 ± 4.3	125.3 ± 17.3<br>p>0.999*	8.6 ± 3.2<br>p>0.999 *<br>p=0.998†	7 ± 2.6<br>p>0.999*<br>p=0.998†	1 ± 1<br>p>0.999*<br>p=0.998†	456 ± 16<br>p=0.331*<br>p=0.682†	504.3 ± 32.1<br>p=0.204*<br>p=0.513†	833 ± 96.3<br>**p=0.001***<br>**p=0.010†**
**TNF-α**	30 ± 5.5	352.6 ± 40.7<br>p=0.708*	54.3 ± 11.5<br>p>0.999 *<br>p=0.784†	52.3 ± 18.7<br>p>0.999*<br>p=0.778†	21 ± 6.2<br>p>0.999*<br>p=0.679†	4875.6 ± 969.5<br>**p<0.001***<br>**p<0.001†**	5929 ± 477.6<br>**p<0.001***<br>**p<0.001†**	3955.6 ± 312.9<br>**p<0.001***<br>**p<0.001†**

*vs. Uninfected, †vs. *M. bovis* BCG

Supplementary Table 6. Cytokines expression (pg/ml) after 12 and 96 hours of ***M. tb* R179** infection of THP-1 cells. The levels of IDO-1, IFN-β, IFN-γ, IL-4, IL-6, IL-8, IL-12p40, IL-12p70, IL-23 and TNF-α were found to be significantly higher after knock-up of IFITs. Although, the levels of IL-1α, IL-1β and IFN-α were found to be similar after knock-up of *IFITs*. After knock-down of IFITs, levels of IDO-1, INF-γ, IL-6, IL-8, IL-12p70, IL-23 and TNF-α were found to be significantly reduced. The levels of IL-1α, IL-1β, IL-4, IL-12p40, IFN-α and INF-β were found to be similar before and after knock-down of *IFITs.* Data presented as mean ± S.D.

**Cytokines**	**Uninfected**	***M. tb* R179**	**Knock-down (siRNA)**			**Knock-up (vector-based over-expression)**		
			IFIT1	***IFIT2***	***IFIT3***	***IFIT1***	***IFIT2***	***IFIT3***
at 12 hours								
**IDO-1**	971 ± 13.4	1338.8 ± 30<br>p=0.902*	0.3 ± 0.5<br>p=0.017*<br>**p<0.001**†	8.6 ± 1.5<br>p=0.019*<br>**p<0.001**†	9 ± 1<br>p=0.019*<br>**p<0.001**†	2849.3 ± 562.2<br>**p<0.001***<br>**p<0.001**†	3148.6 ± 87.6<br>**p<0.001***<br>**p<0.001**†	3693.6 ± 511.2<br>**p<0.001***<br>**p<0.001**†
**IFN-α**	29 ± 1	77.3 ± 2.5<br>p>0.999*	0.3 ± 0.5<br>p>0.999*<br>p>0.999†	6 ± 1<br>p>0.999*<br>p>0.999†	12.6 ± 2<br>p>0.999*<br>p>0.999†	739.3 ± 64.6<br>p=0.206*<br>p=0.287†	574.3 ± 40.5<br>p=0.544*<br>p=0.659†	836.3 ± 114.3<br>p=0.093*<br>p=0.141†
**IFN-β**	0	0<br>p>0.999*	0<br>p>0.999*<br>p>0.999†	0<br>p>0.999*<br>p>0.999†	0<br>p>0.999*<br>p>0.999†	8143.6 ± 271.5<br>**p<0.001***<br>**p<0.001**†	5759.6 ± 781.3<br>**p<0.001***<br>**p<0.001**†	6483.6 ± 158.5<br>**p<0.001***<br>**p<0.001**†
**IFN-γ**	527.3 ± 12	927.3 ± 84<br>p=0.855*	0<br>p=0.587*<br>**p=0.028**†	0<br>p=0.587*<br>**p=0.028**†	0<br>p=0.587*<br>**p=0.028**†	7077.3 ± 583<br>**p<0.001***<br>**p<0.001**†	7868.3 ± 221.9<br>**p<0.001***<br>**p<0.001**†	7563.3 ± 1206.4<br>**p<0.001***<br>**p<0.001**†
**IL-4**	7.6 ± 4.7	800 ± 50.8<br>p=0.106*	0<br>p>0.999*<br>p=0.099†	0.3 ± 0.5<br>p>0.999*<br>p=0.099†	2.3 ± 2.5<br>p>0.999*<br>p=0.102†	5379.6 ± 143.6<br>**p<0.001***<br>**p<0.001**†	8436.3 ± 453.5<br>**p<0.001***<br>**p<0.001**†	4521.3 ± 285.2<br>**p<0.001***<br>**p<0.001**†
**IL-6**	239.3 ± 19.8	918 ± 19.6<br>p=0.257*	0.6 ± 0.5<br>p= 0.990*<br>**p=0.032**†	9 ± 8.1<br>p=0.992*<br>**p=0.032**†	0.6 ± 1.1<br>p=0.992*<br>**p=0.035**†	6532.6 ± 470.2<br>**p<0.001***<br>**p<0.001**†	5303.3 ± 329.7<br>**p<0.001***<br>**p<0.001**†	7818.3 ± 291.9<br>**p<0.001***<br>**p<0.001**†
**IL-8**	657 ± 19.6	8822.3 ± 972.2<br>**p<0.001***	794 ± 32<br>p>0.999*<br>**p<0.001**†	1066.3 ± 74.1<br>p=0.804*<br>**p<0.001**†	1664.6 ± 187.3<br>**p=0.012***<br>**p<0.001**†	6573.6 ± 204.5<br>**p<0.001***<br>**p<0.001**†	6886.6 ± 50.6<br>**p<0.001***<br>**p<0.001**†	2778.6 ± 2096.4<br>**p<0.001***<br>**p<0.001**†

*vs. Uninfected, †vs. *M. tb* R179

**IL-12p40**	94.6 ± 6.6	245 ± 13.5<br>p>0.999[*]	3.6 ± 3.2<br>p>0.999[*]<br>p=0.990[†]	9.3 ± 4.5<br>p>0.999[*]<br>p=0.992[†]	0<br>p>0.999[*]<br>p=0.989[†]	3463.6 ± 458<br>**p<0.001**[*]<br>**p<0.001**[†]	3054.3 ± 858<br>**p<0.001**[*]<br>**p<0.001**[†]	4108 ± 35.5<br>**p<0.001**[*]<br>**p<0.001**[†]
**IL-12p70**	16.6 ± 4.1	302.6 ± 17.7<br>p=0.974[*]	0<br>p>0.999[*]<br>**p=0.001**[†]	3.3 ± 5.7<br>p>0.999[*]<br>**p=0.001**[†]	0<br>p>0.999[*]<br>**p=0.001**[†]	4589.6 ± 54<br>**p<0.001**[*]<br>**p<0.001**[†]	4112 ± 61.7<br>**p<0.001**[*]<br>**p<0.001**[†]	2766.6 ± 201.6<br>**p<0.001**[*]<br>**p<0.001**[†]
**IL-23**	1245 ± 52.7	2009.3 ± 94.4<br>p=0.135[*]	332.3 ± 27.7<br>**p=0.034**[*]<br>**p<0.001**[†]	350.6 ± 12.6<br>**p=0.041**[*]<br>**p<0.001**[†]	346.3 ± 43<br>**p=0.039**[*]<br>**p<0.001**[†]	6610.3 ± 521.1<br>**p<0.001**[*]<br>**p<0.001**[†]	7477.6 ± 848.2<br>**p<0.001**[*]<br>**p<0.001**[†]	7405 ± 489<br>**p<0.001**[*]<br>**p<0.001**[†]
**IL-1α**	243.3 ± 9.4	249.3 ± 41.4<br>p>0.999[*]	71.3 ± 9.4<br>p=0.998[*]<br>p=0.998[†]	68 ± 20.6<br>p=0.999[*]<br>p=0.998[†]	77 ± 11.1<br>p>0.999 [*]<br>p=0.945[†]	702.3 ± 156.7<br>p=0.744[*]<br>p=0.757[†]	601.3 ± 90.1<br>p=0.914[*]<br>p=0.921[†]	438.6 ± 24.1<br>p=0.997[*]<br>p=0.998[†]
**IL-1β**	22 ± 4.3	337.6 ± 23.4<br>p=0.954[*]	39 ± 7.9<br>p>0.999[*]<br>p=0.966[†]	66 ± 12<br>p>0.999[*]<br>p=0.980[†]	10.3 ± 7.3<br>p>0.999[*]<br>p=0.945[†]	757.6 ± 38.2<br>p=0.169[*]<br>p=0.821[†]	697 ± 53.8<br>p=0.264[*]<br>p=0.912[†]	647 ± 24<br>p=0.362[*]<br>p=0.959[†]
**TNF-α**	30 ± 5.5	1222.3 ± 30.7<br>**p=0.001**[*]	30.3 ± 4.5<br>p>0.999[*]<br>**p=0.001**[†]	61 ± 17.5<br>p>0.999[*]<br>**p=0.002**[†]	17 ± 7<br>p>0.999[*]<br>**p=0.001**[†]	5956 ± 281.6<br>**p<0.001**[*]<br>**p<0.001**[†]	8084 ± 411.6<br>**p<0.001**[*]<br>**p<0.001**[†]	9036.6 ± 839.7<br>**p<0.001**[*]<br>**p<0.001**[†]

[*]vs. Uninfected, [†]vs. *M. tb* R179

Cytokines	Uninfected	*M. tb* R179	Knock-down (siRNA)			Knock-up (vector-based over-expression)		
			IFIT1	*IFIT1*	*IFIT2*	*IFIT1*	*IFIT1*	*IFIT2*
at 96 hours								
**IDO-1**	971 ± 13.4	1798 ± 63.6<br>**p=0.008**[*]	0.3 ± 0.5<br>**p=0.001**[*]<br>**p<0.001**[†]	8.6 ± 1.5<br>**p=0.001**[*]<br>**p<0.001**[†]	9 ± 1<br>**p=0.001**[*]<br>**p<0.001**[†]	2445.3 ± 368.3<br>**p<0.001**[*]<br>**p=0.089**[†]	2313.6 ± 52.2<br>**p<0.001**[*]<br>**p=0.316**[†]	2680.6 ± 324.5<br>**p<0.001**[*]<br>**p=0.003**[†]
**IFN-α**	29 ± 1	71.3 ± 12.5<br>p>0.999[*]	0.3 ± 0.5<br>p>0.999[*]<br>p>0.999[†]	6 ± 1<br>p>0.999[*]<br>p>0.999[†]	12.6 ± 2<br>p>0.999[*]<br>p>0.999[†]	127.3 ± 26<br>p>0.999[*]<br>p>0.999[†]	275.6 ± 44.2<br>p>0.999[*]<br>p=0.989[†]	262.6 ± 37<br>p>0.999[*]<br>p=0.990[†]
**IFN-β**	0	6 ± 3.4<br>p>0.999[*]	0<br>p>0.999[*]<br>p>0.999[†]	12<br>p>0.999[*]<br>p>0.999[†]	6<br>p>0.999[*]<br>p>0.999[†]	6347 ± 302<br>**p<0.001**[*]<br>**p<0.001**[†]	5513.3 ± 100.8<br>**p<0.001**[*]<br>**p<0.001**[†]	4776 ± 112.2<br>**p<0.001**[*]<br>**p<0.001**[†]
**IFN-γ**	527.3 ± 12	1014.6 ± 62.6<br>p=0.389[*]	0<br>p=0.287[*]<br>**p<0.001**[†]	0<br>p=0.287[*]<br>**p<0.001**[†]	0<br>p=0.287[*]<br>**p<0.001**[†]	5772 ± 899.6<br>**p<0.001**[*]<br>**p<0.001**[†]	4627.7 ± 522.8<br>**p<0.001**[*]<br>**p<0.001**[†]	4709 ± 138.1<br>**p<0.001**[*]<br>**p<0.001**[†]
**IL-4**	7.6 ± 4.7	517.6 ± 54.8<br>p=0.329[*]	0<br>p>0.999[*]<br>p=0.310[†]	0.3 ± 0.5<br>p>0.999[*]<br>p=0.310[†]	2.3 ± 2.5<br>p>0.999[*]<br>p=0.310[†]	4120.6 ± 390.4<br>**p<0.001**[*]<br>**p<0.001**[†]	4591 ± 715.7<br>**p<0.001**[*]<br>**p<0.001**[†]	5585 ± 542.5<br>**p<0.001**[*]<br>**p<0.001**[†]

[*]vs. Uninfected, [†]vs. *M. tb* R179

**IL-6**	239.3 ± 19.8	2347.6 ± 315.4<br>**p<0.001***	0.6 ± 0.5<br>p=0.965*<br>**p<0.001**†	9 ± 8.1<br>p=0.970*<br>**p<0.001**†	0.6 ± 1.1<br>p=0.965*<br>**p<0.001**†	6645.6 ± 538.5<br>**p<0.001***<br>**p<0.001**†	7180.3 ± 480.4<br>**p<0.001***<br>**p<0.001**†	3915.3 ± 1243<br>**p<0.001***<br>**p<0.001**†
**IL-8**	657 ± 19.6	9531.7 ± 572.8<br>**p<0.001***	889 ± 161<br>p=0.970*<br>**p<0.001**†	548 ± 91.5<br>p>0.999 *<br>**p<0.001**†	991.7 ± 38.2<br>p=0.821*<br>**p<0.001**†	4049 ± 643.8<br>**p<0.001***<br>**p<0.001**†	4932.7 ± 52.5<br>**p<0.001***<br>**p<0.001**†	4684.3 ± 226.6<br>**p<0.001***<br>**p<0.001**†
**IL-12p40**	94.7 ± 6.6	254.7 ± 11.6<br>p=0.997*	3.7 ± 3.2<br>p>0.999*<br>p=0.963†	9.3 ± 4.5<br>p>0.999*<br>p=0.963†	0<br>p>0.999*<br>p=0.963†	2475.7 ± 454.5<br>**p<0.001***<br>**p<0.001**†	2732 ± 421.9<br>**p<0.001***<br>**p<0.001**†	2837.7 ± 820<br>**p<0.001***<br>**p<0.001**†
**IL-12p70**	16.7 ± 4.1	95.3 ± 7.5<br>p>0.999*	0<br>p>0.999*<br>p>0.999†	0.3 ± 0.5<br>p>0.999*<br>p>0.999†	0<br>p>0.999*<br>p>0.999†	3419 ± 153.5<br>**p<0.001***<br>**p<0.001**†	3627.7 ± 185.2<br>**p<0.001***<br>**p<0.001**†	3172.7 ± 725.4<br>**p<0.001***<br>**p<0.001**†
**IL-23**	1245 ± 52.7	267.7 ± 36.6<br>p=0.993*	49 ± 54.5<br>**p<0.001***<br>**p<0.001**†	34 ± 7<br>**p<0.001***<br>**p<0.001**†	38.7 ± 40<br>**p<0.001***<br>**p<0.001**†	724 ± 295.7<br>**p<0.001***<br>**p<0.001**†	536.3 ± 329.8<br>**p<0.001***<br>**p<0.001**†	528.3 ± 376.6<br>**p<0.001***<br>**p<0.001**†
**IL-1α**	243.3 ± 9.4	267.7 ± 46.4<br>p>0.999*	49 ± 4.5<br>p=0.989*<br>p=0.979†	34 ± 4.3<br>p=0.983 *<br>p=0.969†	38.7 ± 9<br>p=0.985*<br>p=0.972†	724 ± 12.5<br>p=0.408*<br>p=0.478†	536.3 ± 86.6<br>p=0.900 *<br>p=0.936†	4541 ± 147.1<br>p=0.910*<br>p=0.945†
**IL-1β**	22 ± 4.3	118.3 ± 27<br>p>0.999*	32.3 ± 2.5<br>p>0.999*<br>p>0.999†	23.3 ± 2.5<br>p>0.999*<br>p>0.999†	52.7 ± 4.7<br>p>0.999*<br>p>0.999†	456.7 ± 15.3<br>p=0.543*<br>p=0.812†	553 ± 96.2<br>p=0.278*<br>p=0.543†	372.3 ± 54.8<br>p=0.783*<br>p=0.952†
**TNF-α**	30 ± 5.5	352.7 ± 40.7<br>p=0.847*	49.3 ± 13.5<br>p>0.999*<br>p=0.884†	21 ± 9.5<br>p>0.999*<br>p=0.827†	24.3 ± 11<br>p>0.999*<br>p=0.835†	6098.7 ± 532<br>**p<0.001***<br>**p<0.001**†	6119 ± 204.4<br>**p<0.001***<br>**p<0.001**†	5411.3 ± 509.1<br>**p<0.001***<br>**p<0.001**†

*vs. Uninfected, †vs. *M. tb* R179

Reference

1. Harper, C. J., Hayward, D., Kidd, M., Wiid, I. & van Helden, P. Glutamate dehydrogenase and glutamine synthetase are regulated in response to nitrogen availability in Myocbacterium smegmatis. *BMC Microbiology* **10**, 138 (2010).
2. Harper, C. J., Hayward, D., Kidd, M., Wiid, I. & van Helden, P. Glutamate dehydrogenase and glutamine synthetase are regulated in response to nitrogen availability in Myocbacterium smegmatis. *BMC Microbiol* **10**, 138 (2010).
3. Viljoen, A. J., Kirsten, C. J., Baker, B., Helden, P. D. van & Wiid, I. J. F. The Role of Glutamine Oxoglutarate Aminotransferase and Glutamate Dehydrogenase in Nitrogen Metabolism in Mycobacterium bovis BCG. *PLOS ONE* **8**, e84452 (2013).
4. Viljoen, A. J., Kirsten, C. J., Baker, B., van Helden, P. D. & Wiid, I. J. F. The Role of Glutamine Oxoglutarate Aminotransferase and Glutamate Dehydrogenase in Nitrogen Metabolism in Mycobacterium bovis BCG. *PLoS One* **8**, (2013).
5. Johnson, R. *et al.* An outbreak of drug-resistant tuberculosis caused by a Beijing strain in the western Cape, South Africa. *Int. J. Tuberc. Lung Dis.* **10**, 1412–1414 (2006).
6. Katibah, G. E. *et al.* tRNA binding, structure, and localization of the human interferon-induced protein IFIT5. *Mol. Cell* **49**, 743–750 (2013).
